# Design, synthesis, docking, ADMET studies, and anticancer evaluation of new 3-methylquinoxaline derivatives as VEGFR-2 inhibitors and apoptosis inducers

**DOI:** 10.1080/14756366.2021.1956488

**Published:** 2021-08-02

**Authors:** Mohammed M. Alanazi, Ibrahim H. Eissa, Nawaf A. Alsaif, Ahmad J. Obaidullah, Wael A. Alanazi, Abdullah F. Alasmari, Hussam Albassam, Hazem Elkady, Alaa Elwan

**Affiliations:** a Department of Pharmaceutical Chemistry, College of Pharmacy, King Saud University, Riyadh, Saudi Arabia; b Pharmaceutical Medicinal Chemistry & Drug Design Department, Faculty of Pharmacy (Boys), Al-Azhar University, Cairo, Egypt; c Department of Pharmacology and Toxicology, College of Pharmacy, King Saud University, Riyadh, Saudi Arabia

**Keywords:** Anticancer, apoptosis, 3-methylquinoxalin, molecular docking, VEGFR-2

## Abstract

Vascular endothelial growth factor receptor-2 (VEGFR-2) plays a critical role in cancer angiogenesis. Inhibition of VEGFR-2 activity proved effective suppression of tumour propagation. Accordingly, two series of new 3-methylquinoxaline derivatives have been designed and synthesised as VEGFR-2 inhibitors. The synthesised derivatives were evaluated *in vitro* for their cytotoxic activities against MCF-7and HepG2 cell lines. In addition, the VEGFR-2 inhibitory activities of the target compounds were estimated to indicate the potential mechanism of their cytotoxicity. To a great extent, the results of VEGFR-2 inhibition were highly correlated with that of cytotoxicity. Compound **27a** was the most potent VEGFR-2 inhibitor with IC_50_ of 3.2 nM very close to positive control sorafenib (IC_50_ = 3.12 nM). Such compound exhibited a strong cytotoxic effect against MCF-7 and HepG2, respectively with IC_50_ of 7.7 and 4.5 µM in comparison to sorafenib (IC_50_ = 3.51 and 2.17 µM). In addition, compounds **28**, **30f**, **30i**, and **31b** exhibited excellent VEGFR-2 inhibition activities (IC_50_ range from 4.2 to 6.1 nM) with promising cytotoxic activity. Cell cycle progression and apoptosis induction were investigated for the most active member **27a**. Also, the effect of **27a** on the level of caspase-3, caspase-9, and BAX/Bcl-2 ratio was determined. Molecular docking studies were implemented to interpret the binding mode of the target compounds with the VEGFR-2 pocket. Furthermore, toxicity and ADMET calculations were performed for the synthesised compounds to study their pharmacokinetic profiles

## 1. Introduction

According to WHO reports, cancer is considered the second major cause of death^1^. By 2030, the incidence of cancer deaths will reach thirteen million worldwide^2^. Although the high advances in the diagnosis and treatment of cancer diseases, the survival of patients remains poor due to the widespread adverse effects of anticancer agents^3^. So that, the discovery of new, effective, selective, and less toxic anticancer agents remains one of the most urgent needs^4^.

Angiogenesis process plays an important role in the growth and regeneration of tissues. Such a role is crucial to prevent ischaemic necrosis and facilitate the survival of the damaged tissues^5^. During the normal state, angiogenesis is controlled by some protein kinases (PKs), which comprise VEGFRs, FGFRs, and EGFRs^6^. PKs can be deregulated under pathological conditions, producing a disturbance in angiogenesis process. This leads to an increasing in the rate of cell division, creating tumour disease^7^.

VEGFRs and their specific agonist (VEGF) are overexpressed in many human tumours, especially solid tumours as gliomas and carcinomas^8^. Therefore, VEGFRs are considered as one the most important regulators of angiogenesis and consequently tumour growth^9^. VEGFRs family comprises three subtypes including VEGFR-1, VEGFR-2, and VEGFR-3^10^. VEGFR-1 controls embryonic vasculogenesis^11^. VEGFR-2 regulates both embryonic vasculogenesis and tumour angiogenesis^12^. On the other hand, VEGFR-3 is responsible for lymphangiogenesis^13^. So that, VEGFR-2 is now the foremost target for antiangiogenic therapy, and its blocking is a relevant approach for the discovery of new drugs against angiogenesis–dependent malignancies^14^. VEGFR-2 inhibitors demonstrated effective suppression of tumour progression. ATP binding site is the main target of most VEGFR-2 inhibitors^15^.

The crystal structures of VEGFR-2 revealed the presence of many pockets at the ATP binding site. (i) Hinge region which locates on the front side and comprises two key amino acid residues (Cys919 and Glu917). These residues participate in H-bond interactions with the adenine ring of ATP. (ii) Gatekeeper region which separates between the hinge region and DFG-motif region. (iii) DFG-motif region which locates on the backside and contains two key amino acids (Glu885 and Asp1046). For maximum activity, VEGFR-2 inhibitors have to bind with these two residues via H-bonds. (iv) Allosteric hydrophobic region which consists of much hydrophobic amino acid residues^16–19^.

VEGFR-2 inhibitors can be classified into three types. Type I inhibitors (ATP competitive inhibitors), e.g. sunitinib **1** can bind to the adenine binding region of the ATP binding site^15^. Type II inhibitors, e.g. sorafenib **2** induce inactive activation of DFG-out confirmation of activation loop. Such type can bind additionally the allosteric hydrophobic pocket of ATP^20^. Type III inhibitors, e.g. vatalanib **3** can form covalent interaction with cysteine amino acid residue at ATP binding site^21^^,^^22^.

**Figure 1.**
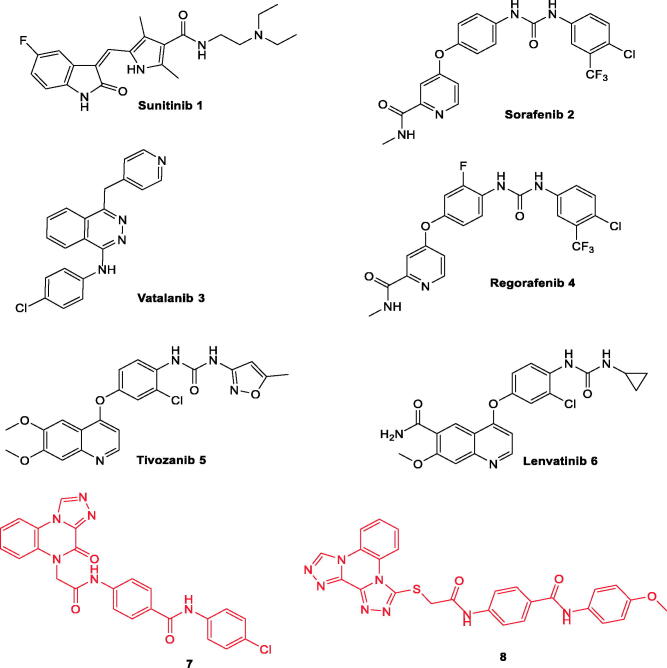
Some clinically used VEGFR-2 inhibitors as well as quinoxaline derivatives having VEGFR-2 inhibitory actions.

Many drugs targeting VEGFR-2 have been approved for clinical use in the treatment of different types of cancers (Figure 1). Sorafenib **2** is a potent VEGFR-2 inhibitor^23^. It is mainly used in the treatment of advanced renal cell carcinoma (RCC) and hepatocellular carcinoma (HCC)^24^. Regorafenib **4,** a fluoro derivative of sorafenib, has been developed to inhibits VEGFR1-3^25^. Tivozanib **5**, a VEGFR-2 inhibitor, has been approved by the FDA in March 2021 for the treatment of RCC^26–28^. Sunitinib **1** is a VEGFR-2 kinase inhibitor that was approved for the treatment of RCC and of gastrointestinal stromal tumours (GIST)^29^.

Binding mode sorafenib, a representative example of VEGFR-2 inhibitors, against VEGFR-2 active site (PDB: 4ASD) was reported in many publications. The heterocyclic (*N*-methylpicolinamide) moiety is buried in the hinge region forming two H-bonds with Cys919 through the N atom of the pyridine ring and NH group of acetamide moiety. In addition, the urea moiety binds to the receptor at the DFG motif through various H-bonding interactions with Glu885 and Asp1046. The terminal phenyl ring occupies the allosteric site forming hydrophobic interactions with the hydrophobic pocket created by the DFG flipping out^30–32^.

Studying the SAR of VEGFR-2 inhibitors and analysing the binding mode of sorafenib revealed that the majority of potent and selective VEGFR-2 inhibitors have four common pharmacophoric features that facilitate their fitting with the active binding pocket^33–35^. The first feature is a flat heteroaromatic ring system (ahead) incorporating at least one H-bond acceptor to interact with the crucial amino acid residue Cys919 in the hinge region^30^^,^^31^. A second feature is a spacer group that gives the inhibitor enough length^36^^,^^37^, making the third feature (pharmacophore) near at DFG motif to bind with two crucial residues (Glu883 and Asp1044)^37^^,^^38^. A pharmacophore is a functional group that comprises at least one H-bond acceptor (HBA) and one H-bond donor (HBD) group (typically amide or urea)^31^^,^^38^. The fourth feature is a terminal hydrophobic moiety that can occupy the created allosteric hydrophobic binding site^16^^,^^39^.

Apoptosis as a form of cellular suicide is one of the important mechanisms by which anticancer agents can affect cancer cells^40^. Apoptosis is regulated by several mediators^41^. Among these are caspases, specifically caspase-3 and caspase-9^42^^,^^43^. Caspase-3 protease is a major mediator of apoptosis catalysing the cleavage of many vital cellular proteins leading to cell death^44^. Caspase-9 activates other executioner caspases as caspase-3, -6, and -7 initiating apoptosis as they cleave several other cellular targets^45^. In addition, the Bcl-2 family proteins are key regulators of apoptosis. This family comprised pro-apoptotic proteins as BAX that promote cell death and anti-apoptotic proteins as Bcl-2 which suppress cell death^46^^,^^47^. The balance between pro-apoptotic and anti-apoptotic proteins (BAX/Bcl-2 ratio) regulates cell fate^48^. Current evidence suggested that inhibition of angiogenesis, anti-angiogenic therapies have been shown to increase apoptosis in tumour cells^49^. VEGFR-2 inhibitors were found to induce and accelerate apoptosis in cancer cells which synergistically potentiates their antitumor effect^30^^,^^49–51^.

Moreover, the literature survey revealed that different scaffolds have been reported as excellent inhibitors of VEGFR-2. These scaffolds comprise quinoline (e.g. lenvatinib **6**^52^), quinazoline^53^, and indazole^54^. Furthermore, quinoxaline, a bioisostere for the aforementioned scaffolds, is considered an important nucleus for anticancer drugs^3^^,^^55–58^. Many quinoxaline derivatives were reported to possess significant VEGFR-2 inhibitory activities^39^^,^^59–62^.

In our previous work, we developed [1,2,4]triazolo[4,3-*a*]quinoxaline containing derivatives as VEGFR-2 inhibitors. Compounds **8** and **9** were the most potent candidates exhibiting an excellent VEGFR-2 inhibitory activity with a promising cytotoxic efficacy against breast and hepatocellular carcinoma^63^^,^^64^. In continuation of our work^63–65^ aimed at synthesising new anticancer agents targeting VEGFR-2 inhibition, new quinoxaline derivatives were designed and synthesised. The synthesised compounds were evaluated for their anti-proliferative activity. In addition, VEGFR-2 inhibitory activities were estimated for all compounds to hint at the potential mechanism of their cytotoxicity. Furthermore, deep investigations were performed on the most active member to assess its effect on apoptotic (caspase-3, caspase-9, and BAX) and anti-apoptotic (Bcl-2) mediators.

### 1.1. Rational of design

**Figure 2.**
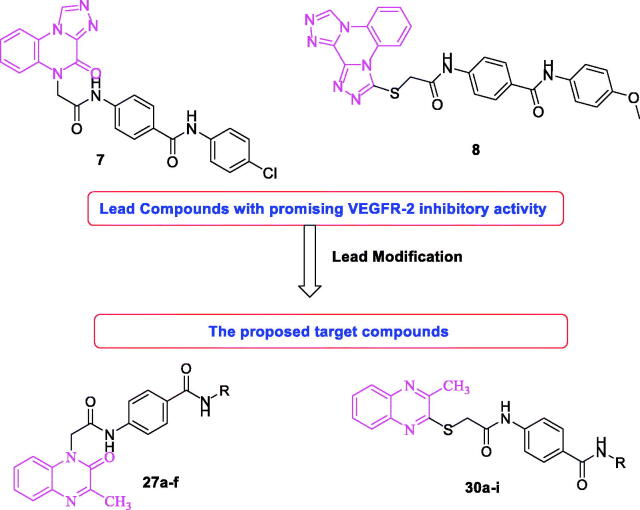
Rational design of new VEGFR-2 inhibitors.

Considering compounds **7** and **8** as leading compounds^63^^,^^64^. The design included the replacement of [1,2,4]triazolo[4,3-*a*]quinoxaline in compound **7** and/or bis([1,2,4]triazolo)[4,3-*a*:3′,4′-*c*]quinoxaline of compound **8** by 3-methylquinoxaline scaffold. 3-Methylquinoxaline is considered as bioisostere for *N*-methylpicolinamide moiety of sorafenib^61^. Two quinoxaline moieties (3-methylquinoxalin-2(1*H*)-one and 3-methylquinoxaline-2-thiol) were used as a biological isostere. As in the lead compounds, *N*-phenylacetamide moiety was utilised as a linker. The pharmacophore (HBD/HBA) was designed to be an amide, diamide, and/or hydrazide groups. Amide pharmacophore served as hydrogen bond donor and acceptor in many reported VEGFR-2 inhibitors^61^^,^^63^^,^^64^^,^^66^. Finally, different, aliphatic, and un/substituted aromatic derivatives were selected to be the terminal hydrophobic moieties to occupy the allosteric hydrophobic pocket (Figure 2).

## 2. Results and discussion

### 2.1. Chemistry

**Scheme 1.**
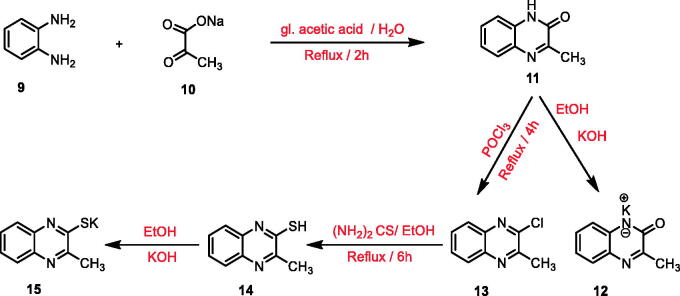
General procedure for preparation of the key potassium salts **12** and **15**.

**Scheme 2.**
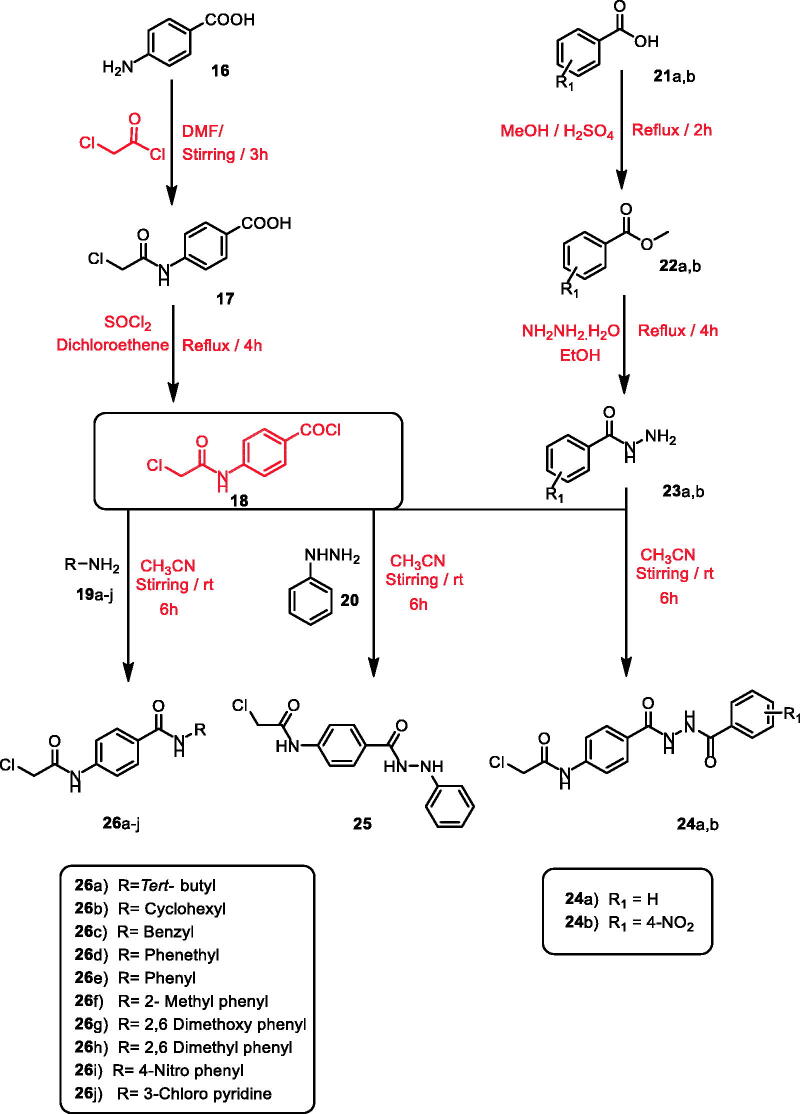
General procedure for preparation of the intermediates **24a**,**b**, **25**, and **26a–j**.

**Scheme 3.**
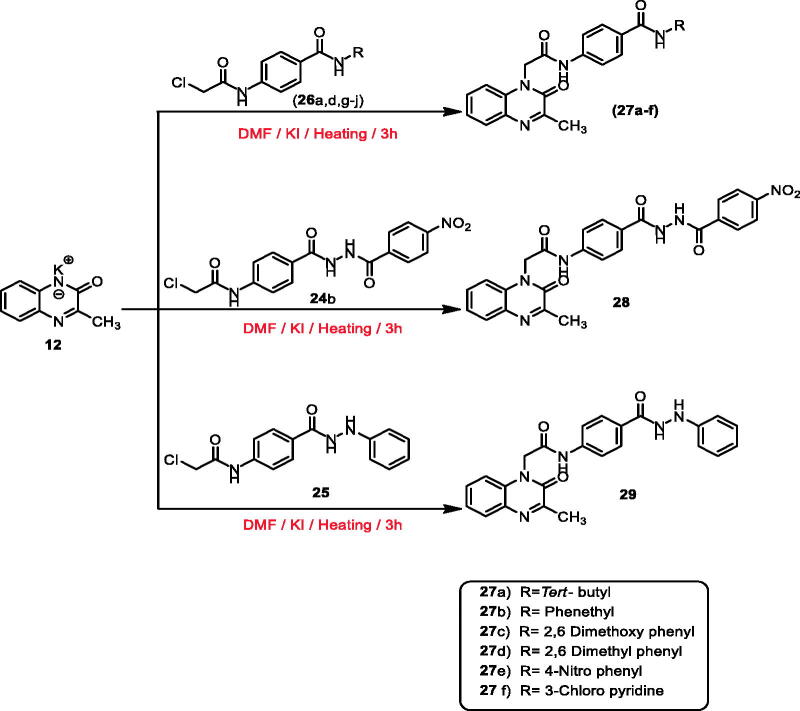
General procedure for preparation of the target compounds **27a–f**, **28**, and **29**.

**Scheme 4.**
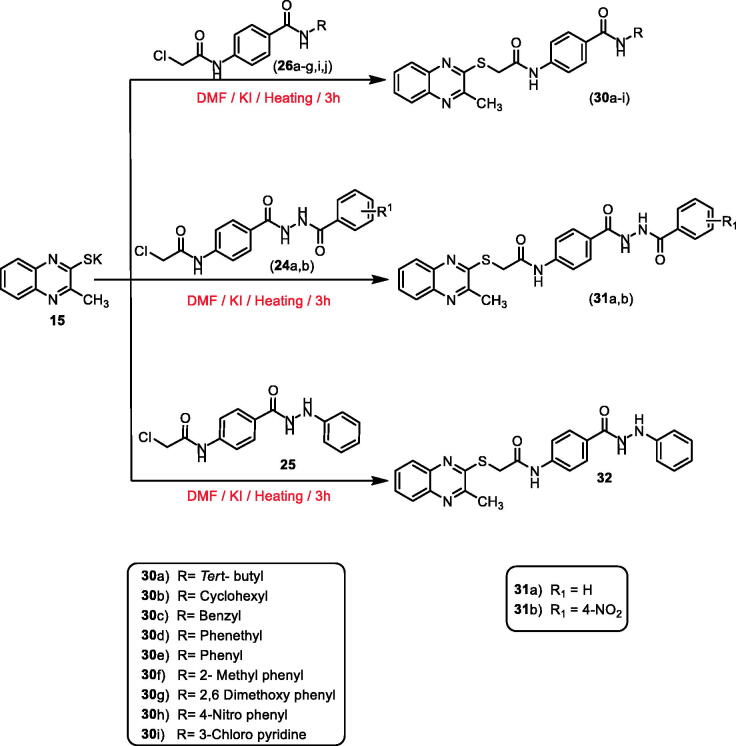
General procedure for preparation of the target compounds **30a–i**, **31a**,**b**, and **32**.

To reach the designed compounds, four Schemes 1–4 were adopted. The synthesis was initiated by the reaction of *o*- phenylenediamine **9** with sodium pyruvate **10** in glacial acetic acid according to the reported procedure to furnish 3-methylquinoxalin-2(1*H*)-one **11**^67^. This starting material **11** was subjected to subsequent treatment with potassium hydroxide to produce the corresponding potassium salt **12**^67^. Chlorination of compound **11** was achieved using phosphorous oxychloride to afford 2-chloro-3-methylquinoxaline **13**^68^. Refluxing the latter with thiourea in absolute ethanol resulted in 3-methylquinoxaline-2-thiol **14**^69^^,^^70^ which was underwent heating with alcoholic potassium hydroxide to provide the corresponding potassium salt **15** (Scheme 1).

According to the literature^71–73^, stirring of commercially available *p*-amino benzoic acid **16** with chloroacetyl chloride in dry DMF in cold conditions yielded 4–(2-chloroacetamido)benzoic acid **17**. Treatment of **17** with thionyl chloride in 1,2 dichloroethane and a catalytic amount of DMF gave the key intermediate 4–(2-chloroacetamido)benzoyl chloride **18**^63^^,^^74^.

On the other hand, methyl esters of appropriate acid derivatives, namely benzoic acid **21a** and 4-nitrobenzoic acid **21b** were prepared by refluxing carboxylic acids in methanol with the presence of sulphuric acid^75^. The ester derivatives **22a**,**b** were treated with hydrazine hydrate to get the corresponding acid hydrazides **23a**,**b**^76^^,^^77^. The produced acid hydrazides **23a**,**b** were then allowed to stir with 4-(2-chloroacetamido)benzoyl chloride **18** in acetonitrile and TEA to furnish the intermediates **24a**,**b**, respectively. Likewise, stirring of phenyl hydrazine **20** with 4-(2-chloroacetamido)benzoyl chloride **18** yielded the corresponding intermediate 2-chloro-*N*-(4–(2-phenylhydrazine-1-carbonyl)phenyl) acetamide **25.** Compound **18** was stirred at room temperature in acetonitrile in the presence of a catalytic amount of TEA with appropriate amines **19a–j** namely, tertiary butyl amine, cyclohexyl amine, benzyl amine, phenethyl amine, aniline, 2-methyl aniline, 2,6 dimethoxy aniline, 2,6 dimethyl aniline, 4-nitro-aniline, and 3-chloropyridine to give the corresponding chloroacetamide intermediates **26a–j**, respectively (Scheme 2).

Synthesis of the final target compounds was illustrated in Schemes 3, 4. The 3-methylquinoxalin-2(1*H*)-one derivative (compounds **27a–f**, **28**, and **29)** were obtained by heating of potassium salt **12** in dry DMF and a catalytic amount of KI with the previously prepared intermediates **26a**,**d**,**g–j**, **24 b**, and **25,** respectively (Scheme 3).

Furthermore, heating of potassium salt **15** with the intermediates **26a–g**,**i**,**j**, **24a**,**b**, and **25** in dry DMF and a catalytic amount of KI afforded the final target compounds **30a–i**, **31a**,**b**, and **32** (methylquinoxaline-2-thiol derivatives) (Scheme 4).

IR spectra of compounds **27a–f** and **30a–i** exhibited the presence of characteristic stretching bands at 3428–3215 cm^−1^ corresponding to NH groups. Furthermore, the NMR spectra of compounds **27a–f** and **30a–i** supported their assigned structures. ^1^H NMR charts of these compounds revealed the appearance of two singlet signals around *δ* 10.5 ppm attributed to the introduced two amidic NH protons. It also demonstrated increased integration of the aromatic protons corresponding to the additional phenyl ring. A characteristic up-field singlet signal equivalent to CH_2_ of acetamide moiety was detected at about *δ* 4.30 ppm. Additionally, a singlet signal of CH_3_ group of 3-methylquinoxalin-2(1*H*)-one moiety appeared around *δ* 2.49 ppm. ^13^C NMR spectra displayed the presence of two peaks at the aliphatic region attributed to the CH_2_ and CH_3_ groups around *δ* 40.0 and 22.5 ppm, respectively.

^1^H NMR spectra of compounds **28**, **29**, **31a**,**b**, and **32** demonstrated the presence of the amidic protons of hydrazide moiety at a range of *δ* 10.02–12.71 ppm. Additionally, ^13^C NMR spectrum of compounds **31b** as an example of these compounds revealed the appearance of two peaks at δ 35.45 and 22.18 ppm corresponding to CH_2_ and CH_3_ groups, respectively. Mass spectroscopic analysis displayed a base peak at 517.0 (*m/z*) corresponding to the exact mass of the compound **31b**.

### 2.2. Biological testing

#### 2.2.1. *In vitro* anti-proliferative activity

*In vitro* cytotoxic activities of the synthesised compounds were evaluated against two different cancer cell lines; breast cancer (MCF-7) and hepatocellular carcinoma (HepG2) using standard MTT colorimetric assay as described by Mosmann^78^^,^^79^. Sorafenib was used as a reference cytotoxic drug. The results of cytotoxicity were expressed as growth inhibitory concentration (IC_50_) values and summarised in Table 1.

**Table 1.** *In vitro* cytotoxicity of the synthesised compounds against MCF-7 and HepG2 cell lines, their VEGFR-2 inhibitory activities on cancer HepG2 cell line, and cytotoxicity for compounds **27a** and **30f** against normal HepG2cell line.

Compounds	Cytotoxicity on cancer cells IC_50_ (µM)^a^		VEGFR-2 IC_50_ (nM)^a^	Cytotoxicity on normal hepatocytes IC_50_ (µM)^a^
	MCF-7	HepG2		
**27a**	7.7	4.5	3.2	19.94
**27b**	62.3	41.5	22.5	NT
**27c**	57.2	42.6	11.6	NT
**27d**	30.7	24.1	21.8	NT
**27e**	42.3	27.5	21.7	NT
**27f**	22.7	19.7	10.7	NT
**28**	17.2	11.7	4.2	NT
**29**	23.5	17.5	9.8	NT
**30a**	61.9	43.7	29.8	NT
**30b**	43.8	34.2	8.7	NT
**30c**	41.9	34.1	13.8	NT
**30d**	42.7	32.4	15.7	NT
**30e**	31.3	24.3	27.4	NT
**30f**	18.1	10.7	4.9	15.73
**30g**	61.7	40.8	38.9	NT
**30h**	21.3	17.7	11.8	NT
**30i**	17.2	12.7	6.1	NT
**31a**	23.1	18.7	10.7	NT
**31b**	19.2	13.7	5.1	NT
**32**	59.5	41.3	23.8	NT
Sorafenib	3.51	2.17	3.12	17.31
Compound **7**	7.2	4.1	3.4	NT
Compound **8**	4.4	3.3	3.2	NT

NT: not tested.

^a^All IC_50_ values are calculated as the mean of at least three different experiments.

General investigation of the cytotoxicity results clarified that the examined compounds had greater anti-proliferative activities against HepG2 than MCF-7 cells. In particular, compound **27a** was found to be the most potent derivative. Such compound showed strong anti-proliferative activities against MCF-7 and HepG2 cancer cell lines with IC_50_ values of 7.7 and 4.5 µM, respectively. These values were close to that of the sorafenib (IC_50_ = 3.51 and 2.17 µM, respectively).

Additionally, compounds **28** (IC_50_ = 17.2 and 11.7 µM), **30f** (IC_50_ = 18.1 and 10.7 µM), **30i** (IC_50_ = 17.2 and 12.7 µM), and **31b** (IC_50_ = 19.2 and 13.7 µM) demonstrated promising cytotoxicity against MCF-7 and HepG2, respectively. Compounds **27f**, **29**, **30h**, and **31a** showed moderate anti-proliferative activities against the two tested cell lines with IC_50_ values ranging from 17.5 to 23.5 µM. While compounds **27d**, **27e**, and **30e** showed moderate activities against only HepG2 cells with IC_50_ values ranging from 24.1 to 27.5 µM.

On the other hand, compounds **30b**, **30c**, and **30d** displayed weak cytotoxic activities against the two cell lines with IC_50_ values ranging from 32.4 to 43.8 µM. While compounds **27d**, **27e**, and **30e** showed weak activities against only MCF-7 with IC_50_ values ranging from 30.7 to 42.3 µM. Compounds **27b**_,_ **27c**, **30a**, **30g**, and **32** showed weak activities against only HepG2 with IC_50_ values ranging from 40.8 to 43.7 µM. In the contrast, these compounds were inactive against the MCF-7 cell line.

Comparing to the lead compounds **7** (IC_50_ = 7.2 and 4.1 µM) and **8** (IC_50_ = 4.4 and 3.3 µM), it was found that the most active compounds **27a** (IC_50_ = 7.2 and 4.1 µM) was slightly less active than these compounds against MCF-7 and HepG2, respectively.

#### 2.2.2. In vitro kinase inhibition assay

The newly prepared compounds have been further assayed for their inhibitory activity towards sorafenib's crucial target (VEGFR-2). The results were reported as 50% inhibition concentration values (IC_50_, expressed as nM) in comparison to sorafenib as a reference drug (Table 1).

To a great extent, the reported results were in good agreement with that of cytotoxicity. This may clarify the possible mechanism of cytotoxic action for the designed compounds. Most of the tested compounds exhibited excellent, moderate, to weak VEGFR-2 inhibitory activities with IC_50_ values ranging from 3.2 to 38.9 nM, compared to positive control sorafenib (IC_50_ = 3.12 nM).

Among them, compound **27a** was the most potent VEGFR-2 inhibitor with an IC_50_ value of 3.2 nM. Besides, compounds **28**, **29**, **30b**, **30f**, **30i**, and **31b** showed strong VEGFR-2 inhibitory activities with IC_50_ values of 4.2, 9.8, 8.7, 4.9, 6.1, and 5.1 nM respectively. Furthermore, compounds **27c**, **27f, 30c**, **30d**, **30h**, and **31a** displayed moderate activities with IC_50_ values ranging from 10.7 to 15.7 nM. Finally, compounds **27b**, **27d**, **27e**, **30a**, **30e**, **30g**, and **32** presented weak activities with an IC_50_ value range of 21.7–38.9 nM.

Comparing to the lead compounds **7** (IC_50_ = 3.4 nM) and **8** (IC_50_ = 3.2 nM), it was found that the most active compounds **27a** (IC_50_ = 3.2 nM) had comparable VEGFR-2 inhibitory activity with these lead compounds.

#### 2.2.3. Cytotoxicity against primary rat hepatocytes (normal hepatic cells)

It is of high importance for anticancer agents to have minimum side effects on normal cells. To assess the selectivity of the synthesised compounds against cancer cells over normal ones, the cytotoxicity of the most active anti-proliferative compounds **27a** and **30f** was evaluated *in vitro* against primary rat hepatocytes using sorafenib as reference^80^. The two compounds **27a** and **30f** showed cytotoxic activity against cancer HepG2 cell line (4.5-fold and 1.5-fold, respectively) more than cytotoxic activity against normal hepatic cells in comparison to sorafenib (8-fold) (Table 1).

#### 2.2.4. Structure-activity relationship

**Figure 3.**
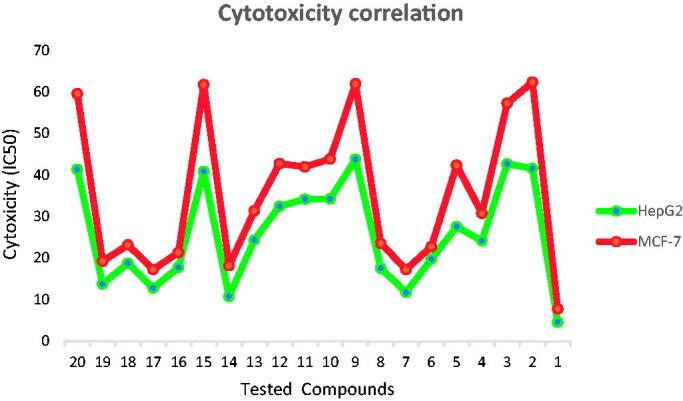
Correlation of cytotoxicity of the synthesised compounds on the two tested cell lines; MCF-7 and HepG2 showing higher sensitivity against HepG2 than MCF-7 cell line.

It was noticed that the synthesised compounds were more effective against HepG2 than the MCF-7 cell line (Figure 3). The obtained data from VEGFR-2 inhibition was highly matched with that of cytotoxicity. So that, the SAR can be built on the results of cytotoxicity and/or VEGFR-2 inhibition. SAR of the newly synthesised compounds was studied along with the pharmacophoric features outlined in the rationale of molecular design.

Firstly, the effect of the flat heteroaromatic ring on biological activity was examined. Comparing the cytotoxicity and VEGFR-2 inhibitory activities of the synthesised compounds incorporating 3-methylquinoxalin-2(1*H*)one moiety (compounds **27a–f**, **28**, and **29**) with that incorporating 3-methylquinoxaline-2-thiol moiety (compounds **30a–i**, **31a**,**b**, and **32**), it was found that 3-methylquinoxalin-2(1*H*)one moiety was more valuable than methylquinoxaline-2-thiol nucleus.

In addition, the impact of pharmacophore (HBD/HBA) moiety was explored. Regarding 3-methylquinoxalin-2(1*H*)one derivatives, it was found that compounds comprising the amide group (compounds **27a–f**) were more active than that containing diamide group (compound **28**), which in turn was more active than that with hydrazide moiety (compound **29**). Concerning for 3-methylquinoxaline-2-thiol series, despite maintaining the same order of activity, it was found that compounds bearing amide (compounds **30a–i**), diamide (compounds **31a,b**), and hydrazide (compound **32**) groups showed decreased activity than that incorporating 3-methylquinoxalin-2(1*H*)one nucleus.

Furthermore, the structure of the terminal hydrophobic tail gave wide varieties of biological activity. In cornering methylquinoxalin-2(1*H*)one derivatives, it was noticed that hydrophobic aliphatic moiety was critical for activity as found in the most potent member **27a** containing tertiary butyl tail.

For the hydrophobic aromatic tail, the heterocyclic ring (compound **27f**) displayed high activity than non-heteroaromatic ones (compounds **27a–d**). Then, the effect of the substitution on the aromatic rings was examined. it was observed that the cytotoxicity and VEGFR-2 inhibitory activities were highly fluctuated by substitution with electron-withdrawing (EWG) and electron-donating (EDG)groups. The unsubstituted phenyl ring (compound **27b**) markedly decreased the biological activity. Substitution with EDG as a 2,6-dimethoxy group (compound **27c**) increased the cytotoxicity. Changing the substitutions to be 2,6-dimethyl groups (compound **27d**) markedly decreased the activity. Additionally, it was found that the cytotoxicity decreased upon substitution with the electron-withdrawing group as in compound **27e**.

For 3-methylquinoxaline-2-thiol derivatives, it was found that aromatic tails (compound **30c-i**) were more effective than aliphatic ones (compounds **30a** and **30b**). For aliphatic derivatives, the bulky tertiary butyl tail (compound **30a**) showed cytotoxic activity higher than the alicyclic one (compound **30b**). About aromatic tail, comparing the IC_50_ of compounds **30f** (2-tolyl derivative), **30h** (4-nitrophenyl derivative), and **30e** (phenyl derivative), indicated that substitution with EDG group was more advantageous than substitution with EWG, which was more favourable than the unsubstituted one. Shifting the 2-tolyl into 5-chloro-2-pyridinyl (hetero-aromatic) moiety (compound **30i**) produced a mild decrease in activity. While changing the 2-tolyl into 2,6-dimethoxy phenyl moiety (compound **30g**) produced a dramatic decrease in activity.

Investigating the activity of compounds **30c, 30d,** and **30e** demonstrated that, insertion of carbon bridge between the phenyl moiety (hydrophobic tail) and the pharmacophore moiety produced an increase in activity with higher priority for a one-carbon bridge (compound **30c**) over than two-carbon one (compound **30d**).

#### 2.2.5. Cell cycle analysis

Tissue homeostasis is tightly controlled by the balance between cell proliferation and cell death^81^. This creates a great link between the cell cycle and apoptosis^82^. Therefore manipulation of the cell cycle efficiently affected apoptotic response^83^.

**Figure 4.**
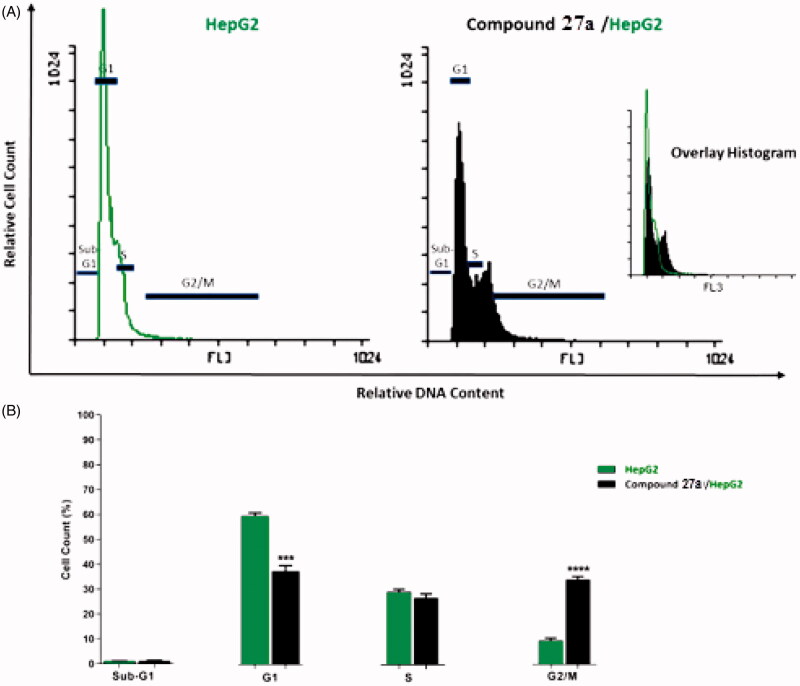
Flow cytometric analysis of cell cycle phases after treatment with compound **27a**. (A) histograms showing the cell cycle distribution of control and treated cells. (B) Column graphs showing the percentage of cells in each phase of the cell cycle.

Flow cytometry analysis as described by Wand et al.^84^^,^^85^ was used to investigate the effect of the most active compound **27a** on the cell cycle progression and apoptosis induction utilising HepG2 cell line. HepG2 cells were treated with 4.5 µM (IC_50_ of compound **27a**) for 24 h, then analysed for its effect on cell cycle distribution. The cell cycle parameters of the incubated cells were compared with untreated control cells (Table 2 and Figure 4).

**Table 2.** Effect of compound **27a** on cell cycle progression in HepG2 cells.

Sample	Cell cycle distribution (%)^a^			
	%Sub-G1	%G1	%S	%G2/M
HepG2	1.13 ± 0.08	59.93 ± 1.16	29.32 ± 1.08	9.62 ± 0.98
Compound ** 27a**/HepG2	1.24 ± 0.17	37.66 ± 2.16***	26.96 ± 1.55	34.14 ± 1.22****

^a^Values are given as mean ± SEM of three independent experiments.

***p < 0.001 and ****p < 0.0001 indicate statistically significant differences from the corresponding control (HepG2) group in unpaired *t*-tests.

The data of flow cytometric analysis revealed the presence of massive accumulation of the treated HepG2 cells at the G2/M (34.14%, 3.5-fold) compared to control cells (9.62%). In addition, a slight difference was observed at the S phase in the percentages of the treated cells (26.96%) and control ones (29.32%). On other hand, the percentage of HepG2 cells increased in the sub G1 phase from 1.13% (control cell) to 1.24% (treated cell). Also, it decreased at the G1 phase from 59.93% (control cell) to 37.66% (treated cell). Such outcomes indicated that compound **27a** had a high ability to hinder cell cycle progression of HepG2 cells at the G2/M phase.

#### 2.2.6. Detection of apoptosis

As compound **27a** produced a high accumulation of HepG2 cells at the G2/M phase, such compound was further investigated for its apoptotic effect using Annexin V and PI double staining assay^86^. In such a procedure, HepG2 cells were treated with compound **27a** at a concentration of 4.5 µM and incubated for 24 h.

**Figure 5.**
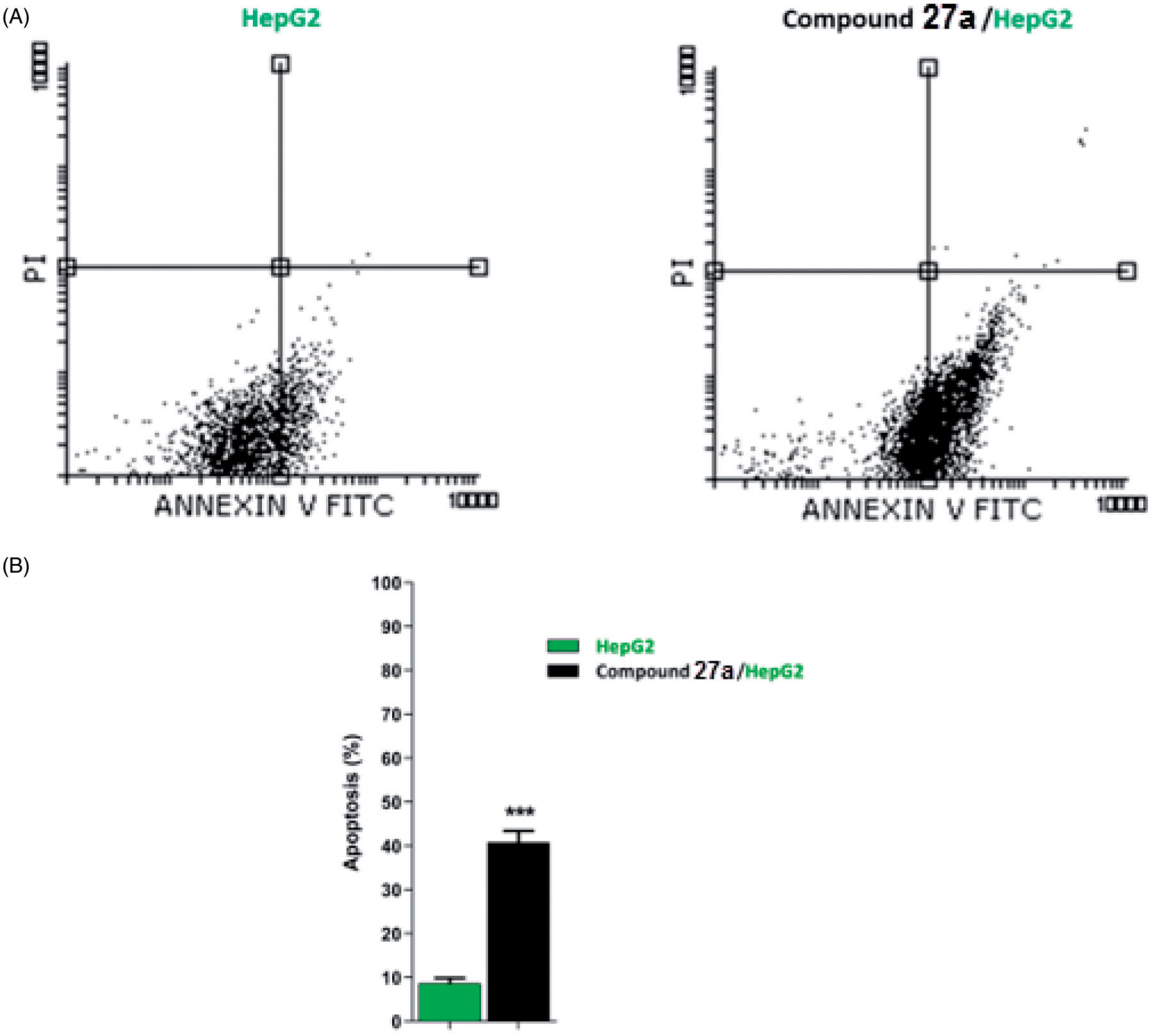
Flow cytometric analysis of apoptosis in HepG2 cells exposed to compound **27a**.

The results revealed that compound **27a** induced an increase of HepG2 cells at early (40.47%) and late (0.35%) stages of apoptosis by about five times more than the untreated cells (8.52 and 0.14%, respectively) (Table 3 and Figure 5).

**Table 3.** Stages of the cell death process in HepG2 cells after treatment with compound **27a**.

Sample	Viable^a^ (left bottom)	Apoptosis^a^		Necrosis^a^ (left top)
		Early (right bottom)	Late (right top)	
HepG2	91.20 ± 1.16	8.52 ± 1.16	0.14 ± 0.01	0.14 ± 0.011
Compound **27a**/HepG2	58.98 ± 2.64	40.47 ± 2.55***	0.35 ± 0.06	0.20 ± 0.04

^a^Values are given as mean ± SEM of three independent experiments.

***p < 0.001 indicates statistically significant difference from the corresponding control (HepG2) group in unpaired *t*-test.

#### 2.2.7. Effects on the levels of active caspase-3 and caspase-9

**Figure 6.**
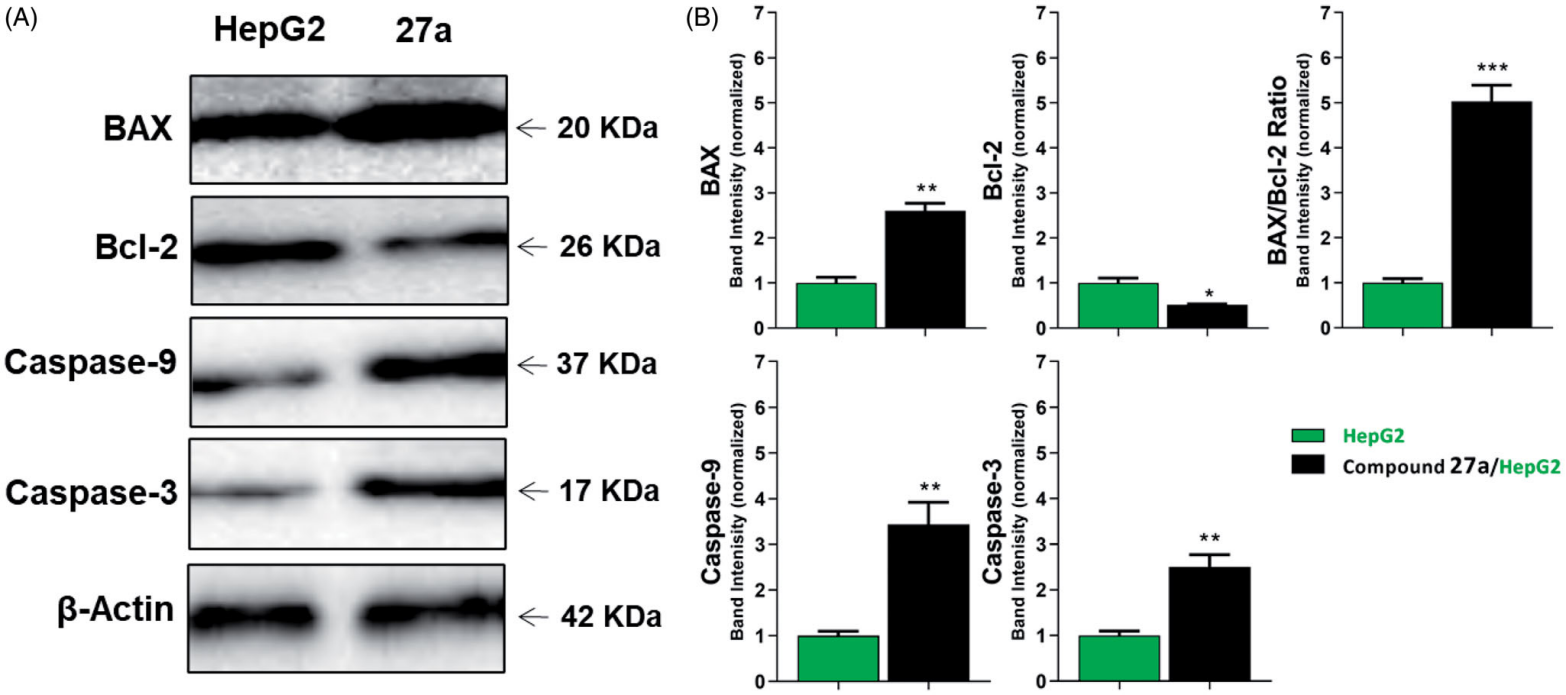
The immunoblotting of caspase-3, caspase-9, BAX, and Bcl-2 (normalised to β-actin). (A) Representative Western blot images show the effect of compound **27a** (at its IC_50_ concentration) on the expression levels of BAX, Bcl-2, active caspases-9, and active caspases-3 proteins in HepG2 cells.

To analyse the effect of the most active compound **27a** on protein expression levels of caspase-3 and caspase-9, Western blot analysis was utilised^87^. In this test, HepG2 cells were treated with compound **27a** at its cytotoxic concentration (4.5 µM) for 24 h. The results displayed a marked increase in the level of caspase-3 (2.5-fold) and caspase-9 (3.43-fold) compared to the control cells (Table 4 and Figure 6). Such findings are consistent with previous reports declared that VEGFR-2 inhibitors can up-regulate both caspase-3 and caspase-9 to induce apoptosis^88^^,^^89^.

**Table 4.** Effect of compound **27a** on the levels of active caspases-3, active caspases-9, BAX, and Bcl-2 proteins in HepG2 cells treated for 24 h.

Sample	Protein expression (normalised to β-actin)^a^				
	Caspases-3	Caspases-9	BAX	Bcl-2	BAX/Bcl-2 ratio
HepG2	1.00 ± 0.10	1.00 ± 0.10	1.00 ± 0.13	1.00 ± 0.11	1.00 ± 0.09
**27a**/HepG2	2.51 ± 0.27**	3.43 ± 0.49**	2.60 ± 0.20**	0.52 ± 0.02*	5.03 ± 0.36***

^a^Values are given as mean ± SEM of three independent experiments.

*p < 0.05, **p < 0.01, ***p < 0.001 indicate statistically significant differences from the corresponding control (HepG2) group in unpaired *t*-tests.

#### 2.2.8. Effects on the levels of Bcl-2 family and BAX/Bcl-2 ratio

In this study, BAX and Bcl-2 expression levels in the HepG2 cell line after the treatment with compound **27a** were determined by quantitative Western blotting. The results revealed that compound **27a** significantly affected the apoptosis pathway in HepG2 cells as it increased the BAX level by 2.6-fold compared to the control cell. Also, it decreased the Bcl-2 level by 2-fold less than the control cells. In addition, the BAX/Bcl-2 ratio was increased 5-fold in the treated cells compared to the control cell (Table 4 and Figure 6). Meanwhile, compound **27a** increased BAX/Bcl-2 ratio, it could trigger apoptosis in the experienced cells^90^^,^^91^.

### 2.3. *In silico* studies

#### 2.3.1. Molecular docking studies

Computational docking studies were carried out against two VEGFR-2 crystal structures (PDB ID; 2OH4 and 4ASD)^92^. Docking studies were performed to explore the binding mode of the synthesised compounds against the ATP binding pocket of VEGFR-2. The docking experiments were carried out using MOE.14 software. Sorafenib was used as a reference molecule. The binding free energies (ΔG) of the tested ligands and sorafenib against each protein were presented in Table 5.

**Table 5.** The calculated ΔG (binding free energies) of the synthesised compounds and reference drug against VEGFR-2 (PDB ID; 2OH4 and 4ASD) (ΔG in Kcal/mole).

Comp.	VEGFR-2 (PDB ID: 2OH4) Δ*G* [Kcal/mole]	VEGFR-2 (PDB ID: 4ASD) Δ*G* [Kcal/mole]
**27a**	−25.71	−28.56
**27b**	−25.45	−25.51
**27c**	−10.82	−11.23
**27d**	−14.22	−20.33
**27e**	−23.61	−22.41
**27f**	−20.71	−21.06
**28**	−26.41	−29.46
**29**	−23.48	−25.22
**30a**	−16.47	−22.47
**30b**	−17.08	−23.62
**30c**	−21.59	−28.38
**30d**	−27.45	−24.17
**30e**	−20.74	−19.11
**30f**	−23.22	−16.83
**30g**	−18.23	−25.34
**30h**	−24.05	−20.07
**30i**	−20.62	−27.93
**31a**	−26.77	−29.15
**31b**	−25.01	−23.61
**32**	−21.66	−23.49
Sorafenib	−30.07	−36.05

**Figure 7.**
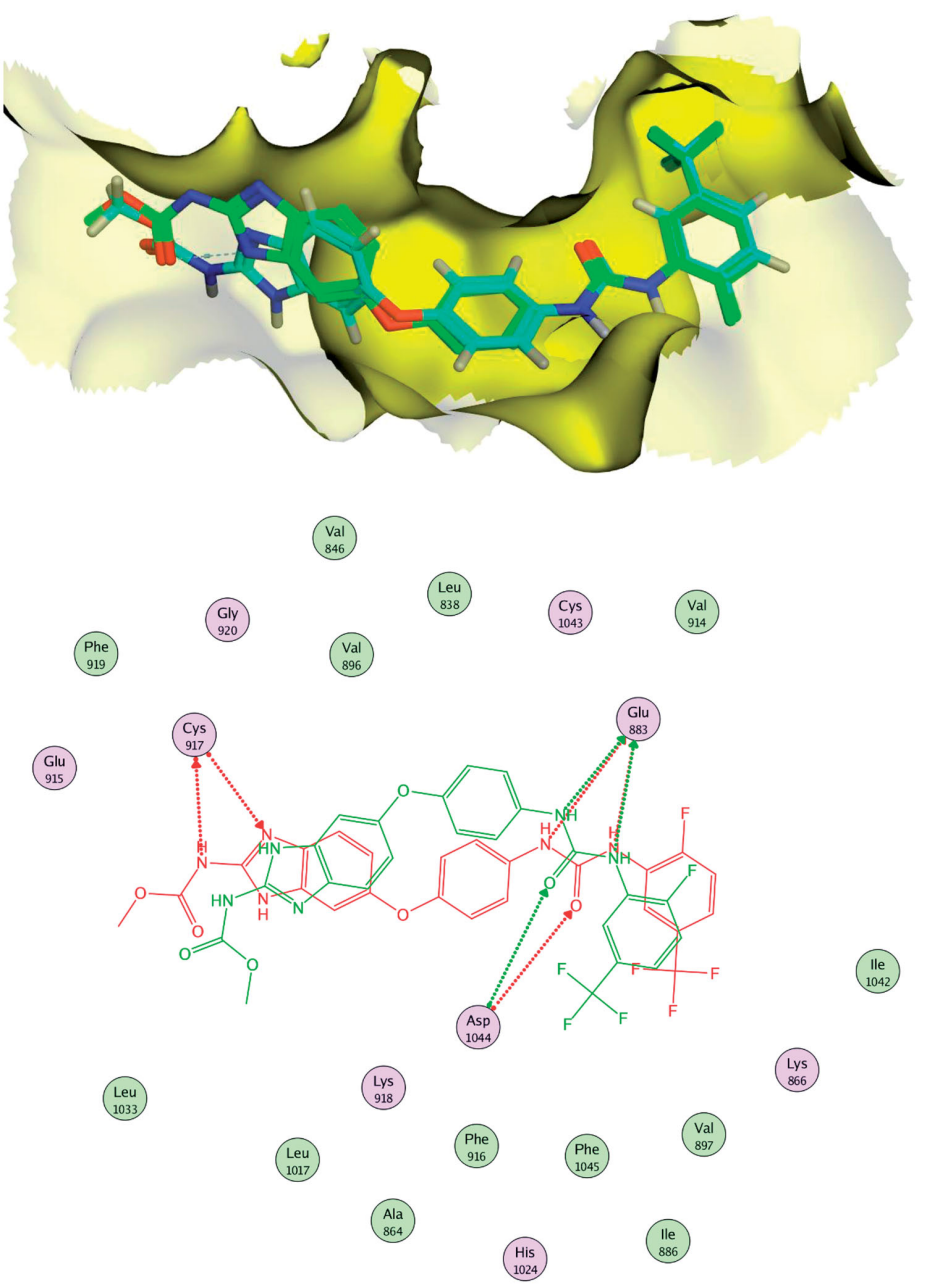
Alignment of the co-crystallised pose and the re-docked pose of the same ligand (PDB ID; 2OH4).

**Figure 8.**
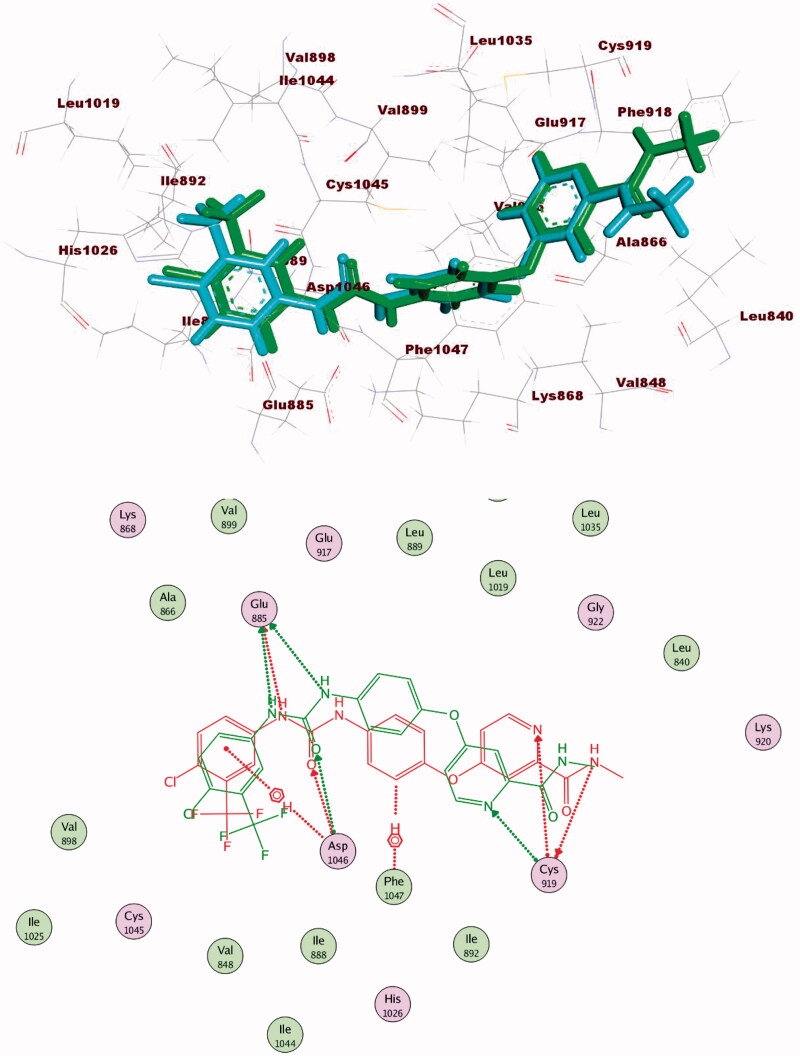
Alignment of the co-crystallised pose and the re-docked pose of the same ligand (PDB ID; 4ASD).

At first, the co-crystallised ligands of each protein were re-docked into the active pockets of VEGFR-2. The resulted RMSD values between the original co-crystallised ligands and the re-docked ones were 1.04 and 1.15 Å. Such values approved the validation of docking processes (Figures 7 and 8).

**Figure 9.**
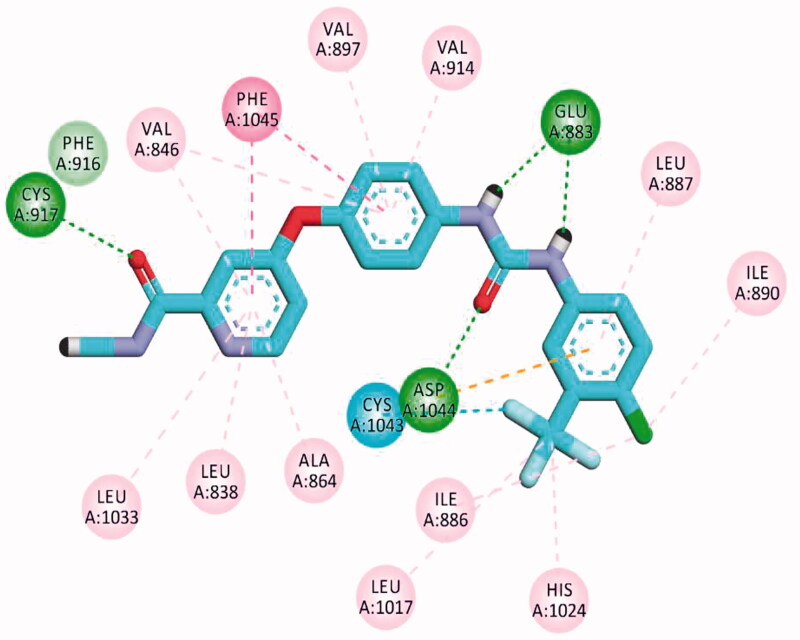
2D binding mode of sorafenib into VEGFR-2 (PDB ID; 2OH4).

**Figure 10.**
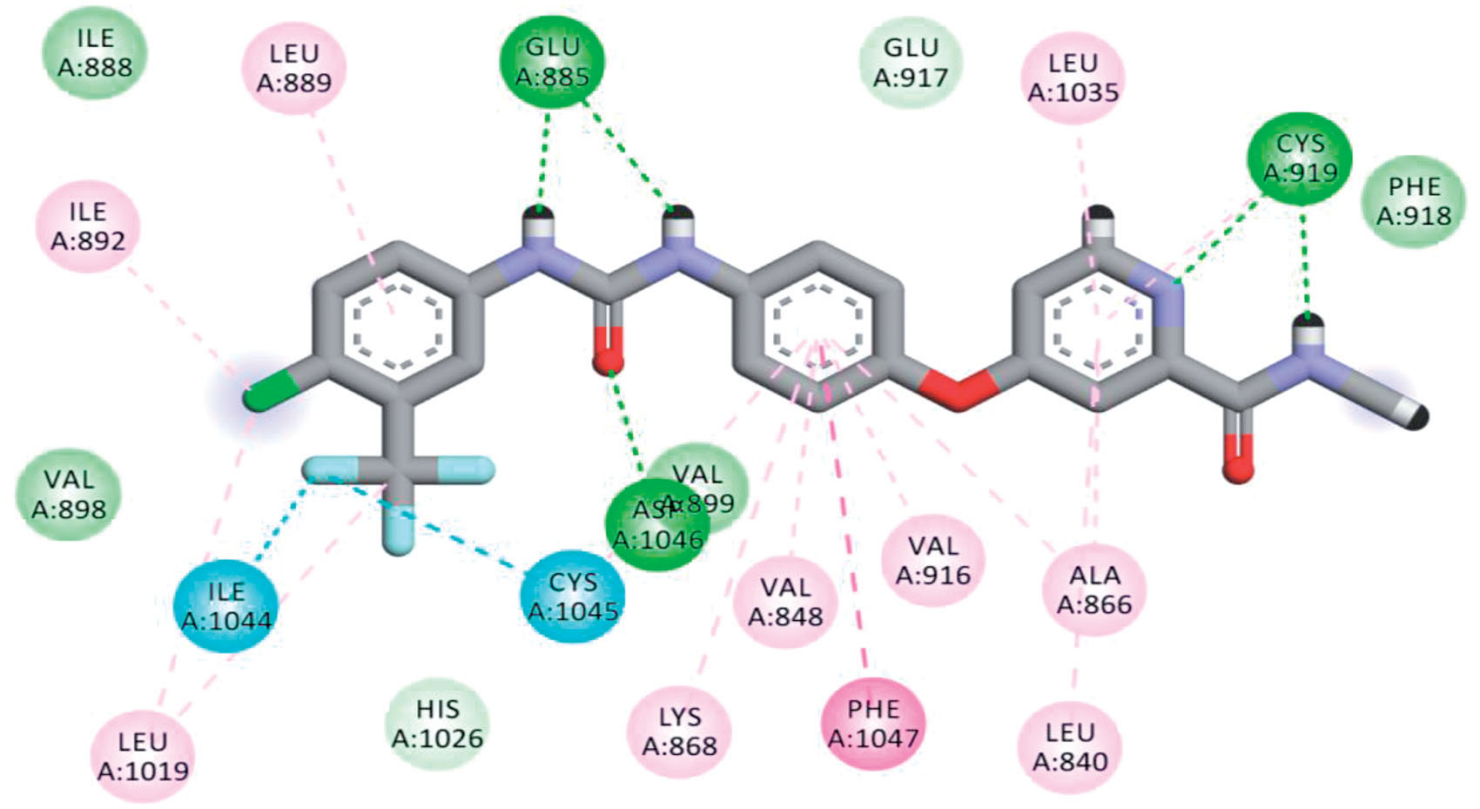
2D binding mode sorafenib into VEGFR-2 (PDB ID; 4ASD).

Docking of sorafenib as a reference compound was performed to compare its binding mode with those of the target compounds. The proposed binding mode of the docked sorafenib was the same in the two pockets. The urea moiety bound the receptor through three hydrogen bonding interactions with the crucial amino acids; Glu883 (Glu885) and Asp1044 (Asp1046). Moreover, the *N*-methylpicolinamide moiety occupied the hinge region, where the pyridine moiety formed one hydrogen bond with Cys917 (Cys919) (Figures 9 and 10).

**Figure 11.**
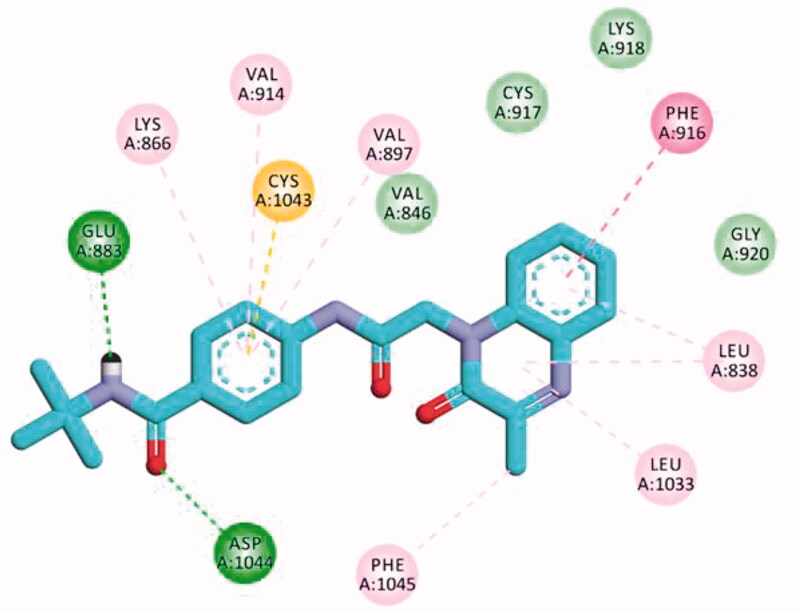
2D binding mode compound **27a** into VEGFR-2 (PDB ID; 2OH4).

The results of docking studies against VEGFR-2 (PDB ID; 2OH4) showed that the tested ligands have binding modes similar to that of sorafenib against the VEGFR-2 active pocket. From each series, the most cytotoxic compound was selected to analyse its biding mode against the active pocket. Compound **27a** as a representative example of 3-methylquinoxalin-2(1*H*)one series revealed an affinity value of −25.71 kcal/mol. The pharmacophore (amide) moiety bound to the key amino acids Asp1046 and Glu388, where the NH group formed a hydrogen bond with Glu388 while the C=O group formed another hydrogen bond with Asp1046. Four hydrophobic interactions took place between the phenyl ring (linker) and the amino acid residues Val897, Cys1043, Val914, and Lys866 in the linker region. Furthermore, the 3-methylquinoxalin-2(1*H*)one moiety occupied the hinge region and was involved in five hydrophobic interactions with Leu838, Leu1033, Phe1045, and Phe916. The *tert*-butyl moiety occupied the allosteric pocket (Figure 11).

**Figure 12.**
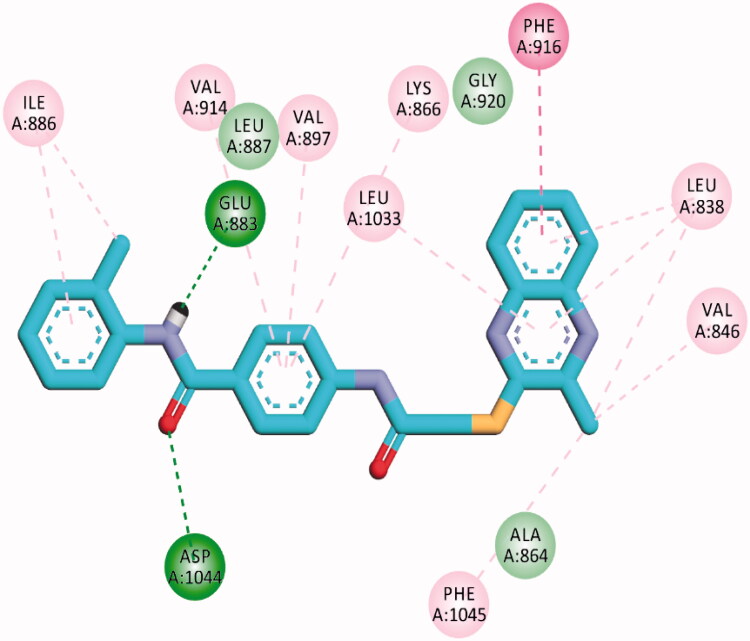
2D binding mode compound **30f** into VEGFR-2 (PDB ID; 2OH4).

The proposed binding mode of **30f** as an example of 3-methylquinoxaline-2-thiol series against VEGFR-2 (PDB ID; 2OH4) was like that of sorafenib. The docking score of such a compound was −23.22 kcal/mol. Compound **30f** interacted with the key amino acid Asp1046 via its hydrogen bond acceptor (C=O) group of amide moiety, while the hydrogen bond donor (NH) group interacted with the carboxylate moiety of Glu388. Three hydrophobic interactions were observed between the spacer phenyl ring and amino acid residues Lys866, Val897, and Val914. In addition, 3-methylquinoxaline-2-thiol moiety formed seven hydrophobic interactions with Phe1045, Val846, Leu838, Phe916, and Leu1033. Finally, the terminal 2-tolyl moiety occupied the allosteric biding site forming two hydrophobic interactions with Ile868 (Figure 12).

**Figure 13.**
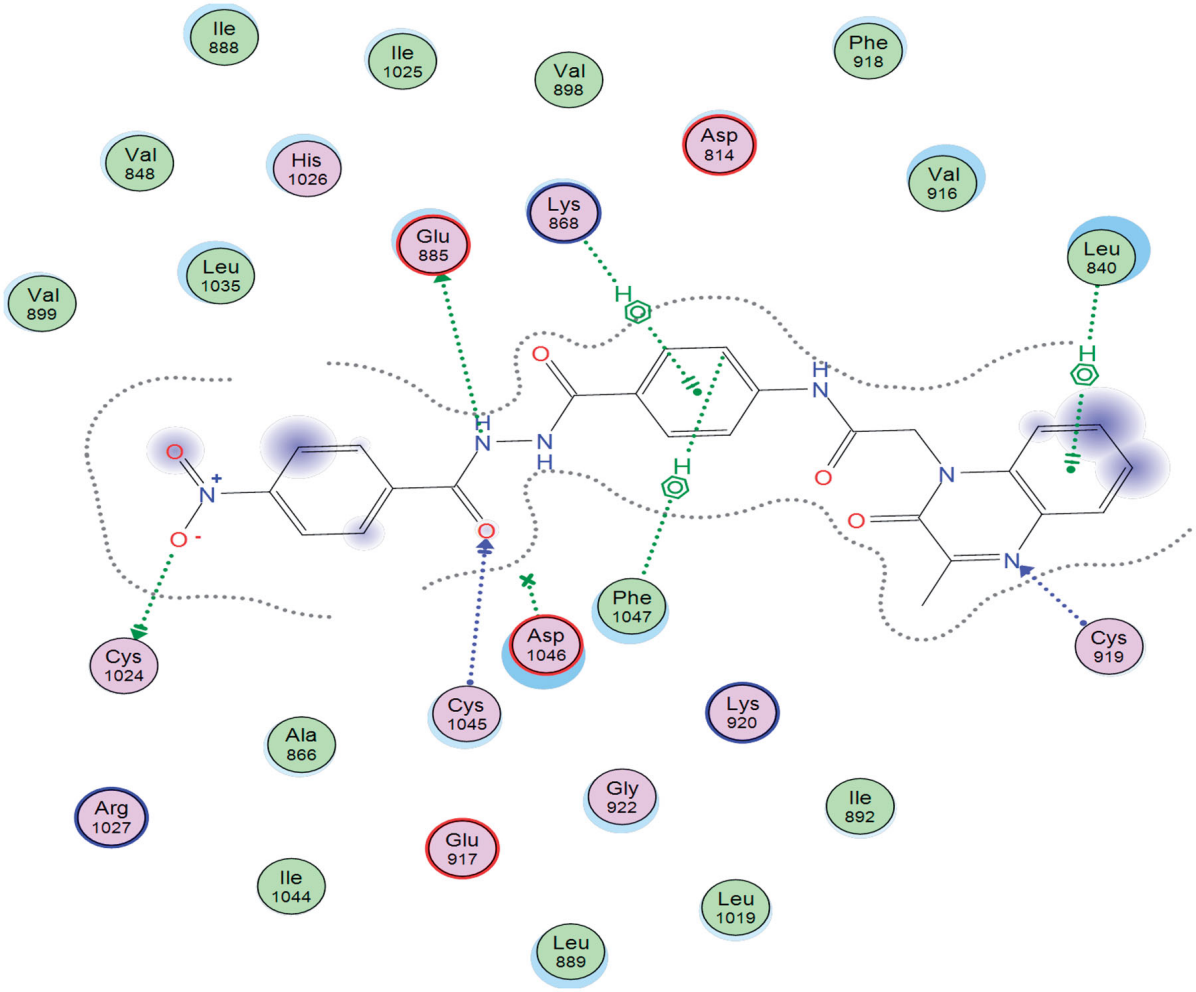
2D binding mode of compound **28** into VEGFR-2. PDB ID; 4ASD.

The proposed binding mode of **28** against VEGFR-2 (PDB ID; 4ASD) was like that of sorafenib. The docking score of such a compound was −29.46 kcal/mol. Compound **28** interacted with Cys1045 via its hydrogen bond acceptor (C=O) group of amide moiety, while the hydrogen bond donor (NH) group interacted with the carboxylate moiety of Glu885. Two hydrophobic interactions were observed between the spacer phenyl ring and amino acid residues Lys868, Phe1047. In addition, 3-methylquinoxaline moiety formed one hydrogen bond with the key amino acid Cys919 and one hydrophobic interaction with Leu840. Finally, the terminal *p*-nitrophenyl moiety occupied the allosteric biding site forming an extra hydrogen bond with Cys1024 (Figure 13).

#### 2.3.2. *In silico* ADMET study

To predict the pharmacokinetics properties of the newly synthesised compounds, computer-aided ADME studies were performed using Discovery Studio 4.0 software. Sorafenib was used as a reference drug. These studies include the assessment of certain parameters as blood-brain barrier (BBB) penetration, absorption level, aqueous solubility, CYP2D6 binding, and plasma protein binding. Predictions of ADME properties for the studied compounds were listed in Supplementary Data).

**Figure 14.**
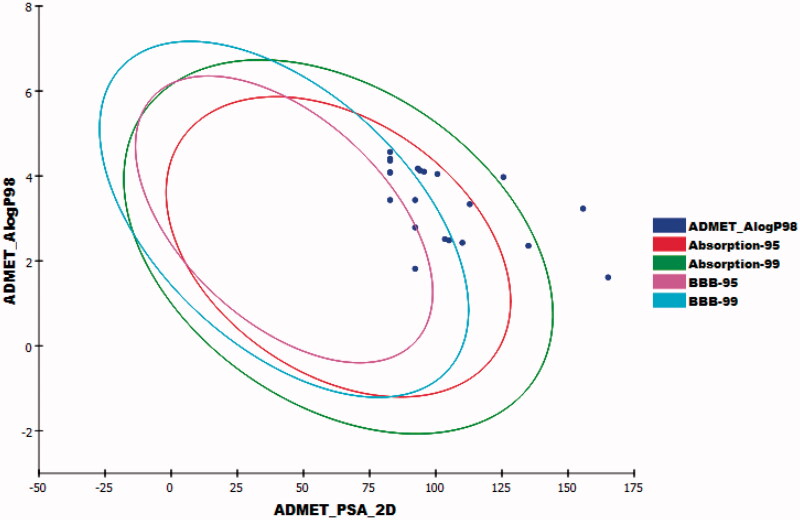
The expected ADMET study for the synthesised compounds.

The results showed that compounds **30a–f** have medium BBB diffusion levels while the rest of the compounds showed low to very low levels. Consequently, the CNS side effects were anticipated to be minimal for the majority of the synthesised compounds. Regarding, aqueous solubility, compounds **27a–c**, **27e**, **28**, and **29** exhibited good levels, while compounds **27d**, **27f**, **30a–i**, **31a**,**b**, and **32** showed poor levels. With respect to absorption parameter, all compounds demonstrated good absorption levels except compounds **27e** showed moderate absorption level and compounds **28**, **30h**, and **31b** which showed poor to very poor intestinal absorption levels. Moreover, the effect on cytochrome P450 2D6 was investigated. The results showed that all the tested compounds were non-inhibitors of CYP2D6. Consequently, hepatotoxicity is not expected upon their administration. The plasma protein binding model displayed that compounds **27a**, **28**, **30a–c**, **30g**,**h**, **31a**,**b**, and **32** were anticipated to bind plasma protein <90%. On the other hand, compounds **27b–f**, **29**, **30d–f**, and **30i** were expected to bind plasma protein more than 90% (Figure 14).

#### 2.3.3. *In silico* toxicity study

The toxicity profile of the synthesised compounds was determined based on the validated and constructed models in Discovery studio 4.0 software^93^^,^^94^. This includes the prediction of certain parameters as FDA rodent carcinogenicity, carcinogenic potency TD_50_, rat maximum tolerated dose, developmental toxicity potential, rat oral LD_50_, rat chronic lowest observed adverse effect level (LOAEL), ocular irritancy, and skin irritancy.

Regarding the FDA rodent carcinogenicity model compounds **27a–f**, **28**, **30b**, **30g**, and **30i** were forecasted to be non-carcinogenic. For carcinogenic potency, TD_50_ mouse model compounds **27a**,**b**, **29**, **30a**, **30c–f**, **30h**, **i**, **31a**,**b**, and **32** showed TD_50_ values ranging from 15,600 to 225,175 mg/kg body weight, which is higher than sorafenib (14,244 mg/kg body weight), while compounds **27c–f**, **28**, **30b**, and **30g** showed carcinogenic potency TD_50_ lower than that of sorafenib. With respect to the rat maximum tolerated dose model (MTD), compounds **29**, **30e**,**f**, **30i**, **31a**, and **32** demonstrated maximum tolerated dose with a range of 0.096 to 0.194 g/kg body weight, which is higher than that of sorafenib **(**0.089 g/kg body weight). In Addition, all compounds were predicted to be non-toxic against the developmental toxicity potential model except compounds **27f**, **30g**, and **30i**. For the rat oral LD_50_ model, all compounds revealed oral LD_50_ values in a range of 1.523 to 19.408 g/kg body weight which is higher than that of sorafenib **(**0.823 g/kg body weight). For the rat chronic LOAEL model, the examined members displayed LOAEL values ranging from 0.036 to 0.376 g/kg body weight. These values are higher than sorafenib (0.005 g/kg body weight). Additionally, all the tested compounds were predicted to be mild irritants against the ocular irritancy model and non-irritant against the skin irritancy model. The values of toxicity parameters were depicted in the Supplementary Data.

## 3. Conclusion

New twenty quinoxaline derivatives were designed and synthesised as anticancer agents with VEGFR-2 inhibitory activity. The synthesised compounds were evaluated *in vitro* for their anti-proliferative activities against breast cancer (MCF-7) and hepatocellular carcinoma (HepG2). Compound **27a** was the most potent derivative revealing strong anti-proliferative activity against MCF-7 and HepG2 cell lines with IC_50_ values of 7.7 and 4.5 µM, respectively, comparing to sorafenib (IC_50_ = 3.51 and 2.17 µM, respectively). In addition, compounds **28** (IC_50_ = 17.2 and 11.7 µM), **30f** (IC_50_ = 18.1 and 10.7 µM), **30i** (IC_50_ = 17.2 and 12.7 µM), and **31b** (IC_50_ = 19.2 and 13.7 µM) demonstrated promising cytotoxicity against MCF-7 and HepG2, respectively. The results of the VEGFR-2 enzyme assay were highly correlated with that of cytotoxicity, where the most potent antiproliferative derivatives exhibited good VEGFR-2 inhibitory effects. Compounds **27a** was the most potent VEGFR-2 inhibitor with an IC_50_ value of 3.2 nM in comparison to sorafenib (IC_50_ = 3.12 nM). SAR revealed that 3-methylquinoxalin-2(1*H*)one moiety was more efficient than 3-methylquinoxaline-2-thiol moiety as a heterocyclic ring. The amide moiety was more advantageous as a pharmacophore than the corresponding diamide and hydrazide moieties. In addition, the tert-butyl moiety was the most effective hydrophobic tail. Cell cycle analysis of compounds **27a** revealed that such compound can arrest HepG2 cell growth at G2/M phase by 3.5-fold greater than control cell. Moreover, compound **27a** induced apoptosis in HepG2 cells by five times more than the control cells. Furthermore, it increased the level of caspase-3 and caspase-9 by 2.5-folds and 3.43-fold, respectively. Also, compound **27a** showed an increase in the BAX level (2.6-fold), decrease in the Bcl-2 level (2-fold), and elevation of BAX/Bcl-2 ratio (5-fold) for the control cell. The results of docking studies revealed that the proposed binding modes of the designed compounds were similar to that of sorafenib. Finally, this work presents compound **27a** as a lead candidate that can be further optimised for the synthesis of a promising VEGFR 2 inhibitor.

## 4. Experimental

### 4.1. Chemistry and material

All the reagents, chemicals, and apparatus were presented in Supplementary Data. Compounds **11**^67^, **12**^67^, **13**^68^, **14**^69^^,^^70^, **15**^69^^,^^70^, and the intermediates (**24a**,**b**, **25**, **26a–j**)^63^^,^^64^ were prepared according to the reported procedures.

#### 4.1.1. General procedure for the synthesis of target compounds 27a–f, 28, and 29

A mixture of potassium salt **12** (0.5 g, 2 mmol) and appropriate intermediates **26a**,**d**,**g–j**, **24b**, and **25** (1 mmol) in dry DMF (15 ml) with the presence of potassium iodide (0.2 g, 1.2 mmol), was heated over a water bath for 3 h. The reaction mixture was then cooled, poured into ice-cooled water (150 ml) with continuous stirring. The solid separated was filtered, washed with water several times, then dried and crystallised from the proper solvent to afford the final target compounds **27a-f**, **28**, and **29**, respectively.

##### 4.1.1.1. N-(tert-butyl)-4-(2-(3-methyl-2-oxoquinoxalin-1(2H)-yl)acetamido)benzamide (27a)

The product was crystallised from EtOH/DCM mixture (50:50) as dark green crystal (0.3 g, 80%); m. p. = 230–232 °C; FT-IR (*ν* max, cm^−1^): 3291 (NH), 3077 (CH aromatic), 2967 (CH aliphatic), 1648 (C=O), 1601 (C=N); ^1^H NMR (700 MHz, DMSO-d6) δ (ppm):10.66 (s, 1H), 7.81–7.79 (m, 3H), 7.64–7.61 (m, 3H), 7.58–7.56 (m, 1H), 7.53 (dd, *J* = 8.5, 1.3 Hz, 1H), 7.38 (s, 1H), 5.16 (s, 2H), 2.49 (s, 3H), 1.38 (s, 9H); ^13^C NMR (176 MHz, DMSO-d6) δ (ppm): 166.00, 165.63, 157.96, 154.85, 141.25, 133.48, 132.46, 131.07, 130.18(2 C), 129.27(2 C), 128.76, 123.92, 118.55(2 C), 115.20, 51.16, 45.76, 29.11(3 C), 21.59; MS *(m/z*): 393.19 (M^+^ +1, base beak, 100%); Anal. Calcd. for C_22_H_24_N_4_O_3_ (392.46): C, 67.33; H, 6.16; N, 14.28; Found C, 67.74; H, 6.32; N, 14.39%.

##### 4.1.1.2. 4-(2-(3-Methyl-2-oxoquinoxalin-1(2H)-yl)acetamido)-N-phenethylbenzamide (27b)

The product was crystallised from EtOH/DCM mixture (50:50) as white crystal (0.34 g, 85%); m. p. = 283–285 °C; FT-IR (*ν* max, cm-1): 3274 (NH), 3038 (CH aromatic), 2921 (CH aliphatic), 1676, 1656, 1630 (C=O), 1604 (C=N); ^1^H NMR (700 MHz, DMSO d6) δ (ppm):10.69 (s, 1H), 10.39 (s, 1H), 8.48 (t, *J* = 5.6 Hz, 1H), 7.82–7.81 (m, 1H), 7.80 (q, *J* = 1.7 Hz, 1H), 7.67–7.62 (m, 2H), 7.57 (ddd, *J* = 8.5, 7.1, 1.5 Hz, 1H), 7.52 (dd, *J* = 8.5, 1.3 Hz, 1H), 7.38 (ddd, *J* = 8.1, 7.1, 1.2 Hz, 1H), 7.30 (tt, *J* = 7.7, 1.7 Hz, 2H), 7.26–7.23 (m, 2H), 7.22–7.19 (m, 1H), 5.16 (s, 2H), 3.47 (ddd, *J* = 8.6, 7.5, 5.9 Hz, 2H), 2.86–2.82 (m, 2H), 2.49 (s, 3H); ^13^C NMR (176 MHz, DMSO-d6) δ (ppm): 165.97, 165.69, 157.97, 154.84, 141.52, 140.03, 133.46, 132.46, 130.18(2 C), 129.28(2 C), 129.11(2 C), 128.81(2 C), 128.56, 126.55, 123.92, 118.79(2 C), 115.19, 45.76, 41.32, 35.64, 21.59; MS (*m/z*): 441.2 (M^+^ +1, 28.68%); Anal. Calcd. for C_26_H_24_N_4_O_3_ (440.50): C, 70.89; H, 5.49; N, 12.72; Found C, 70.99; H, 5.13; N, 12.64%.

##### 4.1.1.3. N-(2,6-Dimethoxyphenyl)-4-(2-(3-methyl-2-oxoquinoxalin-1(2H)-yl)acetamido)-benzamide (27c)

The product was crystallised from ethanol/gl.acetic acid mixture (80:20) as yellow crystal (0.31 g, 80%); m. p. < 300 °C; FT-IR (*ν* max, cm^−1^): 3422, 3293 (NH), 3058 (CH aromatic), 2929 (CH aliphatic), 1661 (C=O), 1602 (C=N); ^1^H NMR (700 MHz, DMSO-d6) δ (ppm):10.76 (s, 1H), 10.52 (s,1H), 7.97 (d, *J* = 6.5 Hz, 1H), 7.94–7.93 (m, 2H), 7.83 (d, *J* = 6.7 Hz, 1H), 7.77 (d, *J* = 8.7 Hz, 2H), 7.73–7.71 (m, 1H), 7.68 (t, *J* = 1.4 Hz, 1H), 7.57 (m, 2H), 7.48 (d*, J* = 1.3 Hz, 1H), 4.33 (s, 2H), 3.32 (s, 6H), 2.68 (s, 3H); ^13^C NMR (176 MHz, DMSO-d6) δ (ppm):167.11, 166.26, 165.46, 155.45, 151.97, 142.71, 140.82, 139.36, 135.19, 131.97, 130.93, 130.38, 130.05, 129.89, 129.05(2 C), 128.91, 128.68, 127.64, 127.43, 127.38, 118.85, 35.46(3 C), 22.19; Anal. Calcd. for C_26_H_24_N_4_O_5_ (472.50): C, 66.09; H, 5.12; N, 11.86; Found C, 66.01; H, 5.15; N, 11.64%.

##### 4.1.1.4. N-(2,6-Dimethylphenyl)-4-(2-(3-methyl-2-oxoquinoxalin-1(2H)-yl)acetamido)-benzamide (27d)

The product was crystallised from EtOH/DCM mixture (50:50) as yellowish white crystal (0.35 g, 85%); m. p. = 240–242 °C; FT-IR (*ν* max, cm^−1^): 3429 (NH), 3067 (CH aromatic), 2934 (CH aliphatic), 1656, (C=O), 1603 (C=N); ^1^H NMR (700 MHz, DMSO-d6) δ (ppm): 10.76 (s, 1H), 9.67 (s, 1H), 7.99 (d, *J* = 8.5 Hz, 2H), 7.81 (dd, *J* = 8.0, 1.5 Hz, 1H), 7.72 (d, *J* = 8.6 Hz, 2H), 7.58 (ddd, *J* = 8.5, 7.0, 1.5 Hz, 1H), 7.54 (dd, *J* = 8.6, 1.3 Hz, 1H), 7.42–7.35 (m, 1H), 7.12 (s, 3H), 5.19 (s, 2H), 2.50 (s, 3H), 2.18 (s, 6H); ^13^C NMR (176 MHz, DMSO-d6) δ (ppm): 165.77, 164.83, 157.98, 154.86, 141.88, 136.12, 135.89, 133.48, 132.48, 130.19(2 C), 129.64(2 C), 129.29(2 C), 129.02(2 C), 128.16, 127.07, 123.94, 118.95, 115.21, 45.79, 21.60, 18.56(2 C); MS (*m/z*): 440.18 (M+, 27.13%); Anal. Calcd. for C_26_H_24_N_4_O_3_ (440.50): C, 70.89; H, 5.49; N, 12.72; Found C, 70.54; H, 5.39; N, 12.52%.

##### 4.1.1.5. 4-(2-(3-Methyl-2-oxoquinoxalin-1(2H)-yl)acetamido)-N-(4-nitrophenyl)benzamide (27e)

The product was crystallised from ethanol/gl.acetic acid mixture (80:20) as brown crystal (0.29 g, 78%); m. p. > 300 °C; FT-IR (*ν* max, cm^−1^): 3428 (NH), 3060 (CH aromatic), 2955 (CH aliphatic), 1653, (C=O), 1599 (C=N); ^1^H NMR (700 MHz, DMSO-d6) δ (ppm): 10.83 (s, 1H), 10.72 (s, 1H), 8.29–8.26 (m, 2H), 8.08–8.06 (m, 2H), 8.02–8.00 (m, 2H), 7.81 (dt*, J* = 8.0, 1.8 Hz, 1H), 7.77–7.74 (m, 2H), 7.59–7.53 (m, 2H), 7.39 (ddd, *J* = 8.2, 7.0, 1.4 Hz, 1H), 5.19 (s, 2H), 2.49 (s, 3H); ^13^C NMR (176 MHz, DMSO-*d*_6_) δ (ppm): 165.92, 157.96, 154.85, 146.10, 142.82, 142.58, 133.46, 130.20(2 C), 129.62(2 C), 129.29(2 C), 129.18(2 C), 125.27(2 C), 123.95, 120.24(2 C), 118.90, 115.21, 45.82, 21.59. Anal. Calcd. for C_24_H_19_N_5_O_5_ (457.45): C, 63.02; H, 4.19; N, 15.31; Found C, 63.17; H, 4.13; N, 15.64%.

##### 4.1.1.6. N-(5-Chloropyridin-2-yl)-4-(2-(3-methyl-2-oxoquinoxalin-1(2H)-yl)acetamido)-benzamide (27f)

The product was crystallised from ethanol/gl.acetic acid mixture (80:20) as dark brown crystal (0.36 g, 90%); m. p. < 300 °C; FT-IR (*ν* max, cm^−1^): 3414 (NH), 3030 (CH aromatic), 2962 (CH aliphatic), 1660 (C=O), 1600 (C=N); ^1^H NMR (700 MHz, DMSO-d6) δ (ppm): 10.78 (s, 1H), 10.21 (s, 1H), 7.97 (d*, J* = 8.4 Hz, 2H), 7.81–7.79 (m, 2H), 7.73 (d, *J* = 8.5 Hz, 2H), 7.58 (td, *J* = 7.6, 6.9, 1.5 Hz, 1H), 7.56–7.53 (m, 1H), 7.40–7.36 (s, 1H), 7.19 (t, *J* = 8.9 Hz, 2H), 5.19 (s, 2H), 2.49 (s, 3H); ^13^C NMR (176 MHz, DMSO-d6) δ (ppm): 165.82, 165.15, 158.00, 157.96, 154.85, 142.05, 136.06, 133.46, 132.48, 130.18, 129.86(2 C), 129.21(2 C), 123.93, 122.62, 122.58, 118.86, 115.68, 115.55, 115.19, 45.80, 21.59; MS (*m/z*): 448.1 (M^+^ +1, base beak, 100%); Anal. Calcd. for C_23_H_18_ClN_5_O_3_ (447.88): C, 61.68; H, 4.05; N, 15.64; Found C, 61.17; H, 4.13; N, 15.36%.

##### 4.1.1.7. 2-(3-Methyl-2-oxoquinoxalin-1(2H)-yl)-N-(4-(2-(4-nitrobenzoyl)hydrazine-1-carbonyl) phenyl)acetamide (28)

The product was crystallised from EtOH/DCM mixture (50:50) as white crystal (0.28 g, 74%); m. p. = 257–259 °C; FT-IR (ν max, cm^−1^): 3271 (NH), 3055 (CH aromatic), 2944 (CH aliphatic), 1656 (C=O), 1601(C=N); ^1^H NMR (700 MHz, DMSO-d6) δ 10.82 (s, 1H), 10.56 (s, 2H), 8.01 (m, 2H), 7.94 (m, 2H), 7.81 (d, *J* = 7.9 Hz, 2H), 7.73 (d, *J* = 8.7 Hz, 2H), 7.56 (dd, *J* = 22.6, 8.2 Hz, 3H), 7.39 (d, *J* = 7.6 Hz, 1H), 5.19 (s, 2H), 2.49 (s, 3H); ^13^C NMR (176 MHz, DMSO-d6) δ 165.86, 164.81 (2C), 157.97, 154.85, 149.86, 142.31, 138.75, 133.45, 132.47(2 C), 130.20 (2C), 129.46(2C), 129.06(3 C), 124.25(2C), 119.01(2C), 115.20, 45.81, 21.59. Anal. Calcd. for C_25_H_20_N_6_O_6_ (500.47): C, 60.00; H, 4.03; N, 16.79; Found C, 60.78; H, 4.54; N, 16.56%.

##### 4.1.1.8. 2-(3-Methyl-2-oxoquinoxalin-1(2H)-yl)-N-(4-(2-phenylhydrazine-1-carbonyl) phenyl) acetamide (29)

The product was crystallised from ethanol/gl.acetic acid mixture (80:20) as greenish yellow crystal (0.3 g, 75%); m. p. < 300 °C; FT-IR (*ν* max, cm^−1^): 3291 (NH), 3041 (CH aromatic), 2967 (CH aliphatic), 1648 (C=O), 1601 (C=N); ^1^H NMR (700 MHz, DMSO-*d*_6_) δ (ppm): 10.70 (s, 1H), 10.31 (s, 1H), 10.02 (s, 1H), 8.33 (dd, *J* = 8.1, 1.4 Hz, 1H), 8.26–7.75 (m, 4H), 7.75–7.72 (m, 2H), 7.72–7.69 (m, 1H), 7.63 (d, *J* = 8.5 Hz, 1H), 7.59–7.56 (m, 1H), 7.49–7.46 (m, 1H), 7.38 (t, *J* = 8.1 Hz, 1H), 7.17–7.14 (m, 1H), 5.22 (s, 2H), 2.74 (s, 3H); ^13^C NMR (176 MHz, DMSO-d6) δ (ppm): 157.97, 155.40, 154.86, 133.47, 132.47(2C), 130.20(3 C), 129.78, 129.29(2C), 129.05(3 C), 128.34(2C), 123.95, 123.50(2C), 115.69, 115.20, 45.79, 21.59; Anal. Calcd. for C_24_H_21_N_5_O_3_ (427.46): C, 67.44; H, 4.95; N, 16.38; Found C, 67.73; H, 4.76; N, 16.54%.

#### 4.1.2. General procedure for the synthesis of target compounds 30a–i, 31a,b, and 32

Equimolar amounts of potassium salt **15** (0.3 g, 1 mmol) and appropriate intermediates **26a–g**,**i**,**j**, **24a**,**b**, and **25** (1 mmol) in dry DMF (15 ml) with the presence of potassium iodide (0.2 g, 1.2 mmol) was heated on a water-bath for 3 h. After cooling, the reaction mixture was poured into an ice-water (150 ml) with continuous stirring. The resulted precipitate was filtered and crystallised from the proper solvent to give the final target compounds **30a–i**, **31a**,**b**, and **32**, respectively.

##### 4.1.2.1. N-(tert-Butyl)-4-(2-((3-methylquinoxalin-2-yl)thio)acetamido)benzamide (30a)

The product was crystallised from ethanol as brown crystal (0.15 g, 70%); m. p. = 231–233 °C; FT-IR (*ν* max, cm^−1^): 3315 (NH), 3048 (CH aromatic), 2969 (CH aliphatic), 1673, 1631 (C=O), 1599 (C=N); ^1^H NMR (700 MHz, DMSO-d6) δ (ppm): 10.64 (s, 1H), 7.97 (d, *J* = 8.2 Hz, 1H), 7.82 (d, *J* = 7.9 Hz, 1H), 7.78 (d, J = 8.3 Hz, 2H), 7.73–7.71 (m, 1H), 7.70–7.68 (m, 1H), 7.67 (d, *J* = 8.3 Hz, 2H), 7.62 (s, 1H), 4.30 (s, 2H), 2.67 (s, 3H), 1.37 (s, 9H); ^13^C NMR (176 MHz, DMSO-d6) δ (ppm): 166.88, 166.11, 155.47, 151.98, 141.71, 140.81, 139.35, 130.97, 130.03(2C), 128.91(2C), 128.72, 128.68(2C), 127.36, 118.51, 51.14, 35.39, 29.11(3 C), 22.19; MS (*m/z*): 409.1 (M^+^ +1, base beak, 100%); Anal. Calcd. for C_22_H_24_N_4_O_2_S (408.52): C, 64.68; H, 5.92; N, 13.71; Found C, 64.56; H, 5.89; N, 13.32%.

##### 4.1.2.2. N-Cyclohexyl-4-(2-((3-methylquinoxalin-2-yl)thio)acetamido)benzamide (30b)

The product was crystallised from ethanol as dark brown crystal (0.18 g, 85%); m. p. = 238–240 °C; FT-IR (*ν* max, cm^−1^): 3289 (NH), 3055 (CH aromatic), 2928, 2851 (CH aliphatic), 1671, 1628 (C=O), 1607 (C=N); ^1^H NMR (700 MHz, DMSO-*d*_6_) δ (ppm): 10.66 (s, 1H), 8.07 (d, *J* = 7.9 Hz, 1H), 7.96 (s, 1H), 7.84–7.81 (m, 3H), 7.71 (ddd, *J* = 8.3, 6.9, 1.6 Hz, 1H), 7.70–7.67 (m, 3H), 4.30 (s, 2H), 3.77–3.72 (m, 1H), 2.67 (s, 3H), 1.83–1.72 (m, 4H), 1.63–1.59 (m, 1H), 1.33–1.27 (m, 4H), 1.12 (s, 1H); ^13^C NMR (176 MHz, DMSO-d6) δ (ppm): 166.92, 165.21, 155.47, 151.98, 141.88, 140.82, 139.36, 130.04(2C), 128.91(2C), 128.68(2C), 127.37, 118.62(2C), 48.71, 35.38, 32.95(2C), 25.75, 25.44(2C), 22.18; MS (*m/z*): 435.1 (M^+^ +1, base beak, 100%); Anal. Calcd. for C_24_H_26_N_4_O_2_S (434.56): C, 66.33; H, 6.03; N, 12.89; Found C, 66.39; H, 6.09; N, 12.98%.

##### 4.1.2.3. N-benzyl-4-(2-((3-methylquinoxalin-2-yl)thio)acetamido)benzamide (30c)

The product was crystallised from ethanol as yellow crystal (0.16 g, 75%); m. p. = 235–237 °C; FT-IR (*ν* max, cm^−1^): 3287 (NH), 3071 (CH aromatic), 2933 (CH aliphatic), 1674, 1633, (C=O), 1607 (C=N); ^1^H NMR (700 MHz, DMSO-*d*_6_) δ (ppm): 10.69 (s, 1H), 8.94 (s, 1H), 7.96 (dd, *J* = 8.0, 1.5 Hz, 1H), 7.88 (d, *J* = 8.5 Hz, 2H), 7.82 (dd, *J* = 8.3, 1.6 Hz, 1H), 7.73–7.70 (m, 3H), 7.68 (td, *J* = 7.6, 1.5 Hz, 1H), 7.32 (d, *J* = 6.3 Hz, 4H), 7.24 (tt, *J* = 5.9, 2.4 Hz, 1H), 4.47 (d, *J* = 5.9 Hz, 2H), 4.31 (s, 2H), 2.67 (s, 3H); ^13^C NMR (176 MHz, DMSO-d6) δ (ppm): 166.97, 166.10, 155.45, 151.97, 142.13, 140.81, 140.27, 139.35, 130.04(2C), 129.49(2C), 128.90(2C), 128.73(2C), 128.69, 128.67, 127.67, 127.38, 127.16, 118.77, 43.02, 35.41, 22.18; MS (*m/z*): 443.1 (M^+^ +1, 79.98%); Anal. Calcd. for C_25_H_22_N_4_O_2_S (442.54): C, 67.85; H, 5.01; N, 12.66; Found C, 67.73; H, 5.09; N, 12.78%.

##### 4.1.2.4. 4-(2-((3-Methylquinoxalin-2-yl)thio)acetamido)-N-phenethylbenzamide (30d)

The product was crystallised from ethanol as brown crystal (0.18 g, 85%); m. p. = 220–222 °C; FT-IR (*ν* max, cm^−1^): 3299 (NH), 3026 (CH aromatic), 2925 (CH aliphatic), 1664, 1630 (C=O), 1607 (C=N); ^1^H NMR (700 MHz, DMSO-d6) δ (ppm): 10.67 (s, 1H), 8.47 (s, 1H), 7.98–7.94 (m, 1H), 7.85–7.79 (m, 3H), 7.72 (ddd*, J* = 8.4, 7.0, 1.7 Hz, 1H), 7.71–7.66 (m, 3H), 7.32–7.27 (m, 2H), 7.26–7.23 (m, 2H), 7.23–7.18 (m, 1H), 4.31 (s, 2H), 3.47 (ddd, *J* = 8.7, 7.5, 5.9 Hz, 2H), 2.84 (t, *J* = 7.5 Hz, 2H), 2.67 (s, 3H); ^13^C NMR (176 MHz, DMSO d6) δ (ppm): 166.95, 166.05, 155.45, 151.97, 141.98, 140.81, 140.04, 139.35, 130.03(2C), 129.76 (2C), 129.11(2C), 128.90, 128.81, 128.68(2C), 128.53, 127.38, 126.54, 118.75, 41.32, 35.66, 35.40, 22.18; MS (*m/z*): 457.2 (M^+^ +1, base beak, 100%); Anal. Calcd. for C_26_H_24_N_4_O_2_S (456.56): C, 68.40; H, 5.30; N, 12.27; Found C, 68.39; H, 5.09; N, 12.32%.

##### 4.1.2.5. 4-(2-((3-Methylquinoxalin-2-yl)thio)acetamido)-N-phenylbenzamide (30e)

The product was crystallised from ethanol as brown crystal (0.18 g, 85%); m. p. = 218–220 °C; FT-IR (*ν* max, cm^−1^): 3268 (NH), 3039 (CH aromatic), 2970, 2908 (CH aliphatic), 1666, 1640 (C=O), 1597 (C=N); ^1^H NMR (700 MHz, DMSO-*d*_6_) δ (ppm): 10.75 (s, 1H), 10.14 (s, 1H), 7.98–7.95 (m, 3H), 7.83 (dd, *J* = 8.0, 1.6 Hz, 1H), 7.80–7.76 (m, 4H), 7.72 (ddd, *J* = 8.3, 6.9, 1.6 Hz, 1H), 7.68 (ddd, *J* = 8.4, 7.0, 1.6 Hz, 1H), 7.37–7.33 (m, 2H), 7.09 (tt, *J* = 7.4, 1.2 Hz, 1H), 4.33 (s, 2H), 2.67 (s, 3H); ^13^C NMR (176 MHz, DMSO-d6) δ (ppm): 167.07, 165.34, 155.44, 151.97, 142.46, 140.82, 139.74, 139.36, 130.02(2C), 129.93, 129.20(2C), 129.04(2C), 128.89, 128.68(2C), 127.37, 123.97, 120.79, 118.79, 35.44, 22.18; MS (*m/z*): 429.1 (M^+^ +1, base beak, 100%); Anal. Calcd. for C_24_H_20_N_4_O_2_S (428.51): C, 67.27; H, 4.70; N, 13.08; Found C, 67.04; H, 4.89; N, 13.42%.

##### 4.1.2.6. 4-(2-((3-Methylquinoxalin-2-yl)thio)acetamido)-N-(o-tolyl)benzamide (30f)

The product was crystallised from EtOH/DCM mixture (50:50) as white crystal (0.2 g, 90%); m. p. = 247–249 °C; FT-IR (*ν* max, cm^−1^): 3262 (NH), 3056 (CH aromatic), 2911 (CH aliphatic), 1659, 1641 (C=O), 1607 (C=N); ^1^H NMR (700 MHz, DMSO-*d*_6_) δ (ppm): 10.75 (s, 1H), 9.77 (s, 1H), 7.97 (dd, *J* = 8.4, 4.1 Hz, 3H), 7.84 (d, *J* = 8.1 Hz, 1H), 7.77 (d, *J* = 8.4 Hz, 2H), 7.73 (t, *J* = 7.5 Hz, 1H), 7.69 (t, *J* = 7.5 Hz, 1H), 7.34 (d, *J* = 7.8 Hz, 1H), 7.27 (d, *J* = 7.5 Hz, 1H), 7.22 (t, *J* = 7.5 Hz, 1H), 7.17 (t, *J* = 7.4 Hz, 1H), 4.33 (s, 2H), 2.68 (s, 3H), 2.24 (s, 3H); ^13^C NMR (176 MHz, DMSO-d6) δ (ppm): 167.05, 165.12, 155.46, 151.98, 142.42, 140.82, 139.36, 136.99, 134.13, 130.75, 130.05, 129.57(2C), 129.14(2C), 128.91, 128.69, 127.38, 127.03, 126.44, 126.33, 118.82, 35.44, 22.19, 18.40; MS (*m/z*): 443.1 (M^+^ +1, base beak, 100%); Anal. Calcd. for C_25_H_22_N_4_O_2_S (442.54): C, 67.85; H, 5.01; N, 12.66; Found C, 67.73; H, 5.09; N, 12.78%.

##### 4.1.2.7. N-(2,6-Dimethoxyphenyl)-4-(2-((3-methylquinoxalin-2-yl)thio)acetamido) benzamide (30g)

The product was crystallised from EtOH/DCM mixture (50:50) as light brown crystal (0.17 g, 80%); m. p. = 258–260 °C; FT-IR (*ν* max, cm^−1^): 3298 (NH), 3033 (CH aromatic), 2931 (CH aliphatic), 1665 (C=O), 1607 (C=N); ^1^H NMR (700 MHz, DMSO-d6) δ (ppm): 10.77 (s, 1H), 9.37 (s, 1H), 7.98 (t, *J* = 8.3 Hz, 4H), 7.93 (d, *J* = 8.1 Hz, 1H), 7.84 (d, *J* = 8.1 Hz, 1H), 7.79 (d, *J* = 8.3 Hz, 2H), 7.75–7.72 (m, 1H), 7.71–7.67 (m, 1H), 7.35–7.33 (m, 1H), 4.34 (s, 2H), 3.99–3.97 (s, 3H), 2.69 (s, 6H); ^13^C NMR (176 MHz, DMSO-d6) δ (ppm): 167.09, 164.78, 155.46, 151.99, 151.84, 142.57, 140.83, 139.37, 137.68, 130.06, 129.45(2C), 129.05, 129.00, 128.92, 128.69, 127.39, 126.88, 124.35, 119.00, 118.90, 110.11, 56.46(2C), 35.46, 22.20; Anal. Calcd. for C_26_H_24_N_4_O_4_S (488.56): C, 63.92; H, 4.95; N, 11.47; Found C, 63.56; H, 4.45; N, 11.56%.

##### 4.1.2.8. 4-(2-((3-Methylquinoxalin-2-yl)thio)acetamido)-N-(4-nitrophenyl)benzamide (30h)

The product was crystallised from ethanol as pale yellow crystal (0.12 g, 60%); m. p. = 200–202 °C; FT-IR (*ν* max, cm^−1^): 3316 (NH), 3028 (CH aromatic), 2921 (CH aliphatic), 1682, 1650 (C=O), 1596 (C=N); ^1^H NMR (700 MHz, DMSO-d6) δ (ppm): 10.76 (s, 1H), 10.26 (s, 1H), 7.96 (dd, *J* = 9.2, 2.3 Hz, 3H), 7.82 (td, *J* = 7.6, 7.0, 1.8 Hz, 3H), 7.82–7.76 (m, 2H), 7.71 (ddd, *J* = 8.3, 6.9, 1.6 Hz, 1H), 7.68 (ddd, *J* = 8.3, 6.9, 1.6 Hz, 1H), 7.43–7.38 (m, 2H), 4.33 (s, 2H), 2.67 (s, 3H); ^13^C NMR (176 MHz, DMSO-d6) δ (ppm): 167.10, 165.42, 155.43, 151.96, 142.61, 140.81, 139.36, 138.73, 130.02, 129.61(3 C), 129.25(2C), 128.96(2C), 128.89, 128.68, 127.55, 127.36, 122.24, 118.80, 35.43, 22.18; Anal. Calcd. for C_24_H_19_N_5_O_4_S (473.51): C, 60.88; H, 4.04; N, 14.79; Found C, 60.39; H, 4.58; N, 14.66%.

##### 4.1.2.9. N-(5-Chloropyridin-2-yl)-4-(2-((3-methylquinoxalin-2-yl)thio)acetamido)benzamide (30i)

The product was crystallised from ethanol as brown crystal (0.19 g, 90%); m. p. = 190–192 °C; FT-IR (*ν* max, cm^−1^): 3274 (NH), 3041 (CH aromatic), 2945 (CH aliphatic), 1679 (C=O), 1599 (C=N); ^1^H NMR (700 MHz, DMSO-d6) δ (ppm): 10.86 (s, 1H), 10.78 (s, 1H), 8.44 (t, *J* = 2.6 Hz, 1H), 8.24–8.23 (m, 1H), 8.04–8.02 (m, 2H), 7.96 (d, *J* = 3.3 Hz, 2H), 7.91 (d, *J* = 4.8 Hz, 2H), 7.82 (d, *J* = 6.6 Hz, 1H), 7.76 (s, 1H), 7.72–7.71 (m, 1H), 4.32 (s, 2H), 2.67 (s, 3H); ^13^C NMR (176 MHz, DMSO-d6) δ (ppm): 167.35, 167.16, 155.42, 151.96, 151.48, 146.72, 140.80, 139.35, 138.27, 130.95, 130.04(2C), 129.69(2C), 128.91, 128.67(2C), 127.38, 118.86, 118.71, 116.18, 35.45, 22.18; MS (*m/z*): 464.1 (M^+^ +1, 68.32%),; Anal. Calcd. for C_23_H_18_ClN_5_O_2_S (463.94): C, 59.54; H, 3.91; N, 15.10; Found C, 59.56; H, 3.59; N, 15.35%.

##### 4.1.2.10. N-(4-(2-benzoylhydrazine-1-carbonyl)phenyl)-2-((3-methylquinoxalin-2-yl)thio) acetamide (31a)

The product was crystallised from ethanol as brown crystal (0.13 g, 65%); m. p. = 195–197 °C; FT-IR (*ν* max, cm^−1^): 3262 (NH), 3038 (CH aromatic), 2921 (CH aliphatic), 1657 (C=O), 1601 (C=N); ^1^H NMR (700 MHz, DMSO-*d*_6_) δ (ppm): 10.76 (s, 1H), 10.48 (s, 1H), 10.42–10.41 (s, 1H), 7.94 (d, *J* = 6.8 Hz, 4H), 7.83 (d, *J* = 6.8 Hz, 1H), 7.77 (d, *J* = 4.6 Hz, 1H), 7.72 (d, *J* = 1.6 Hz, 1H), 7.69–7.67 (m, 1H), 7.60 (d, *J* = 6.1 Hz, 2H), 7.53 (m, 3H), 4.33 (s, 2H), 2.68 (s, 3H); ^13^C NMR (176 MHz, DMSO-d6) δ (ppm): 167.11, 166.36, 165.77(2C), 155.45, 151.97, 142.68, 140.82, 139.36, 133.09, 132.32, 130.05(2C), 129.00(3 C), 128.98(2C), 128.68(2C), 127.93, 127.38, 118.90, 35.45, 22.19; MS (*m/z*): 472.1 (M^+^ +1, 53.18%); Anal. Calcd. for C_25_H_21_N_5_O_3_S (471.54): C, 63.68; H, 4.49; N, 14.85; Found C, 63.88; H, 4.68; N, 14.99%.

##### 4.1.2.11. 2-((3-Methylquinoxalin-2-yl)thio)-N-(4-(2-(4-nitrobenzoyl)hydrazine-1-carbonyl) phenyl) acetamide (31b)

The product was crystallised from ethanol as brownish red crystal (0.15 g, 70%); m. p. = 196–198 °C; FT-IR (*ν* max, cm^−1^): 3261 (NH), 3044 (CH aromatic), 2911 (CH aliphatic), 1664 (C=O), 1601 (C=N); ^1^H NMR (700 MHz, DMSO-*d*_6_) δ (ppm): 10.81 (s, 2H), 10.55 (s, 1H), 8.39 (t, *J* = 8.7 Hz, 4H), 8.17 (s, 2H), 7.96 (d, *J* = 8.1 Hz, 1H), 7.93 (d, *J* = 8.4 Hz, 1H), 7.84 (d, *J* = 3.8 Hz, 1H), 7.78 (d, *J* = 8.3 Hz, 1H), 7.72 (t, *J* = 7.6 Hz, 1H), 7.68 (t, *J* = 7.5 Hz, 1H), 4.33 (s, 2H), 2.67 (s, 3H); ^13^C NMR (176 MHz, DMSO-d6) δ (ppm): 165.70, 164.87, 164.85, 164.75, 155.44, 149.98, 149.89, 139.36, 138.67, 130.08(2C), 129.52(2C), 129.47(3 C), 127.38(3 C), 124.32(2C), 119.10, 118.93, 35.45, 22.18; MS (*m/z*): 517.0 (M^+^ +1, base beak, 100%); Anal. Calcd. for C_25_H_20_N_6_O_5_S (516.53): C, 58.13; H, 3.90; N, 16.27; Found C, 58.21; H, 3.98; N, 16.48%.

###### 4.1.2.12. 2-((3-Methylquinoxalin-2-yl)thio)-N-(4-(2-phenylhydrazine-1-carbonyl)phenyl) acetamide (32)

The product was crystallised from ethanol as white crystal (0.18 g, 85%); m. p. = 185–187 °C; FT-IR (*ν* max, cm^−1^): 3272 (NH), 3083 (CH aromatic), 2931 (CH aliphatic), 1676 (C=O), 1598 (C=N); ^1^H NMR (700 MHz, DMSO-d6) δ (ppm): 12.71 (s, 1H), 10.76 (s, 1H), 10.20 (s, 1H), 7.92–7.88 (m, 2H), 7.84–7.79 (m, 2H), 7.76–7.73 (m, 2H), 7.69–7.65 (m, 2H), 7.38–7.31 (m, 2H), 7.15 (t, *J* = 7.7 Hz, 1H), 6.78 (d, *J* = 12.6, 8.0, 7.3 Hz, 2H), 4.32 (s, 2H), 2.69 (s, 3H); ^13^C NMR (176 MHz, DMSO-d6) δ (ppm):155.42, 151.96, 143.54, 140.81, 139.35, 131.08, 130.96, 130.03(2C), 129.79(3 C), 129.19(3 C), 128.9(2C)0, 119.77, 118.91, 118.87, 112.78, 112.01, 35.45, 22.18; Anal. Calcd. for C_24_H_21_N_5_O_2_S (443.53): C, 64.99; H, 4.77; N, 15.79; Found C, 64.54; H, 4.79; N, 15.88%.

### 4.2. Biological testing

#### 4.2.1. *In vitro* anti-proliferative activity

The *in vitro* antiproliferative activities were assessed using the MTT assay protocol^78^^,^^79^^,^^95^. As shown in Supplementary Data.

#### 4.^2^.^2^. *In vitro* VEGFR-2 enzyme inhibition assay

The synthesised compounds were estimated for their *in vitro* inhibition on human VEGFR-2 in HepG2 cell line; using ELISA kit^96^ as described in Supplementary Data.

#### 4.^2^.^3^. Flow cytometry analysis for cell cycle

Cell cycle analysis for the most potent candidate **27a** has been carried out through Flow cytometric analysis as described in Supplementary Data^84^^,^^85^.

#### 4.2.4. Flow cytometry analysis for apoptosis

Apoptosis analysis for compound **27a** in HepG2 cells was carried out using Annexin V-FITC as shown in Supplementary Data^30^^,^^86^.

#### 4.2.5. Determination of active caspase-3 and caspase-9 levels

Quantitative assay of caspase-3 and caspase-9 activation was performed using Caspase- Invitrogen Caspase-3 ELISA Kit (KHO1091) and Invitrogen Caspase 9 Human ELISA Kit (BMS2025)^87^^,^^97–99^ as shown in Supplementary Data.

### 4.^3^. *In silico* studies

#### 4.3.1. Docking studies

The docking studies were performed utilising MOE.14 software as described in Supplementary Data^57^^,^^100^^,^^101^. The final figures were visualised using Discovery studio 4.0^102^.

#### 4.3.2. ADMET study

ADMET descriptors were determined using Discovery studio 4.0. as described in Supplementary Data^101^^,^^103^.

#### 4.3.3. Toxicity study

The toxicity parameters were calculated using Discovery studio 4.0. as described in Supplementary Data^102^.
